# Systemic antibiotics for *Pseudomonas aeruginosa* infection in outpatients with non-hospitalised exacerbations of pre-existing lung diseases: a randomised clinical trial

**DOI:** 10.1186/s12931-024-02860-9

**Published:** 2024-06-06

**Authors:** Josefin Eklöf, Imane Achir Alispahic, Karin Armbruster, Therese Sophie Lapperre, Andrea Browatzki, Rikke Holmen Overgaard, Zitta Barrella Harboe, Julie Janner, Mia Moberg, Charlotte Suppli Ulrik, Helle Frost Andreassen, Ulla Møller Weinreich, Jakob Lyngby Kjærgaard, Jenny Villadsen, Camilla Sund Fenlev, Torben Tranborg Jensen, Christina Wellendorph Christensen, Jette Bangsborg, Christian Ostergaard, Khaled Saoud Ali Ghathian, Alexander Jordan, Tobias Wirenfeldt Klausen, Thyge Lynghøj Nielsen, Torgny Wilcke, Niels Seersholm, Pradeesh Sivapalan, Jens-Ulrik Stæhr Jensen

**Affiliations:** 1 Department of Internal Medicine, Herlev Gentofte University Hospital, Section of Respiratory Medicine, Copenhagen University Hospital, Herlev Gentofte, Hellerup, Denmark; 2 Department of Respiratory Medicine and Infectious Diseases, Copenhagen University Hospital, Bispebjerg Frederiksberg, Denmark; 3 Department of Respiratory Medicine, Antwerp University Hospital, Antwerp, Belgium; 4 Laboratory of Experimental Medicine and Pediatrics, University of Antwerp, Antwerp, Belgium; 5 Department of Respiratory Medicine and Infectious Diseases, Copenhagen University Hospital, North Zealand, Denmark; 6 Department of Respiratory Medicine, Copenhagen University Hospital, Hvidovre, Denmark; 7 Department of Respiratory Medicine, Aalborg University Hospital and Department of Clinical Medicine, Aalborg, Denmark; 8 Department of Internal Medicine, Hospital of Southwest Jutland, Esbjerg, Denmark; 9 Department of Clinical Microbiology, Copenhagen University Hospital, Herlev, Denmark; 10 Department of Clinical Microbiology, Copenhagen University Hospital, Hvidovre, Denmark; 11 Department of Clinical Medicine, University of Copenhagen, Copenhagen, Denmark

## Abstract

**Background:**

The effect of dual systemic antibiotic therapy against *Pseudomonas aeruginosa* in patients with pre-existing lung disease is unknown. To assess whether dual systemic antibiotics against *P. aeruginosa* in outpatients with COPD, non-cystic fibrosis (non-CF) bronchiectasis, or asthma can improve outcomes.

**Methods:**

Multicenter, randomised, open-label trial conducted at seven respiratory outpatient clinics in Denmark. Outpatients with COPD, non-CF bronchiectasis, or asthma with a current *P. aeruginosa*-positive lower respiratory tract culture (clinical routine samples obtained based on symptoms of exacerbation not requiring hospitalisation), regardless of prior *P. aeruginosa-*status, no current need for hospitalisation, and at least two moderate or one hospitalisation-requiring exacerbation within the last year were eligible. Patients were assigned 1:1 to 14 days of dual systemic anti-pseudomonal antibiotics or no antibiotic treatment. Primary outcome was time to prednisolone or antibiotic-requiring exacerbation or death from day 20 to day 365.

**Results:**

The trial was stopped prematurely based in lack of recruitment during the COVID-19 pandemic, this decision was endorsed by the Data and Safety Monitoring Board. Forty-nine outpatients were included in the study. There was a reduction in risk of the primary outcome in the antibiotic group compared to the control group (HR 0.51 (95%CI 0.27–0.96), *p* = 0.037). The incidence of admissions with exacerbation within one year was 1.1 (95%CI 0.6–1.7) in the dual antibiotic group vs. 2.9 (95%CI 1.3–4.5) in the control group, *p* = 0.037.

**Conclusions:**

Use of dual systemic antibiotics for 14 days against *P. aeruginosa* in outpatients with chronic lung diseases and no judged need for hospitalisation, improved clinical outcomes markedly. The main limitation was the premature closure of the trial.

**Trial Registration:**

ClinicalTrials.gov, NCT03262142, registration date 2017–08-25.

**Supplementary Information:**

The online version contains supplementary material available at 10.1186/s12931-024-02860-9.

## Summary of the article’s main point

This is the first randomised controlled trial to report that dual systemic anti-pseudomonal antibiotic treatment seems to be a well-tolerated and effective treatment for reducing exacerbations in patient with chronic lung disease and a *Pseudomonas aeruginosa*-positive lower airway sample culture.

## Introduction

*Pseudomonas aeruginosa*is a Gram-negative bacterium that is associated with a considerable burden of symptoms, hospitalisation and death in patients with chronic pulmonary diseases, including COPD, non-cystic fibrosis (CF) bronchiectasis, and asthma [1–3].

Currently, clinical practice relies on data from observational studies, suggesting clinical benefits of systemic antibiotic treatment in patients with pre-existing lung disease, including findings extrapolated from the treatment of *P. aeruginosa*infections in children with cystic fibrosis, where dual systemic anti-pseudomonal therapy has become a key treatment [4–8]. Based on these studies, international guidelines for patients with bronchiectasis recommend targeted antibiotic interventions against *P. aeruginosa*, ranging from monotherapy to combination therapy [7]. For COPD and asthma, no recommendations have been made, probably based on the lack of available clinical data.

Thus, current recommendations rely on low grade evidence, and there is a need for clinical trial data to clarify whether systemic antibiotics, including dual treatment, against *P. aeruginosa* in patients with chronic pulmonary diseases can improve clinical outcomes. We therefore conducted a randomised, good clinical practice (GCP) monitored, controlled trial to determine whether dual systemic antibiotics against *P. aeruginosa* in patients with COPD, non-CF bronchiectasis, or asthma, and no current indication for hospital admission, can reduce antibiotic or prednisolone requiring exacerbations and death. We hypothesised that antibiotic treatment would lower the risk of exacerbations and mortality in outpatients with COPD, non-CF bronchiectasis, or asthma and a *P. aeruginosa* positive lower respiratory tract sample.

## Methods

### Study design and participants

The study is a multicenter, randomised, GCP monitored, controlled, open-label trial conducted in outpatients with COPD, non-CF bronchiectasis, or asthma with a *P. aeruginosa*-culture positive lower respiratory tract sample. The study was carried out at seven respiratory outpatient clinics in Denmark between October 2017, and March 2023. Outpatients with a *P. aeruginosa*-positive lower respiratory tract sample (sputum, tracheal secretion, bronchial secretion or bronchial alveolar lavage) obtained within the previous 30 days, regardless of prior *P. aeruginosa*-status, and with a physician-judged no need for hospitalisation, were systematically screened and consecutively invited to participate if they fulfilled inclusion criteria and no exclusion criteria (Additional file 1: study protocol). The study was approved by the Ethics Committees (H-15010949), the Danish Medicines Agency (EudraCT 2015–003399-58) and the Danish Data Protection Agency (HGH-2017–036), and was monitored by a national GCP unit. The trial is registered at ClinicalTrials.gov (NCT03262142). No financial incentive was provided to the investigators or participants.

Outpatients were randomly assigned 1:1 to either systemic dual antibiotic treatment (antibiotic group) or no antibiotic treatment (control group) and stratified by study site and age (≤ 70 years vs. > 70 years) (see Appendix for details regarding randomization sequence). The antibiotic intervention consisted of 14 days of combination therapy with piperacillin/tazobactam 4/0.5 g, administered intravenously four times daily, and oral ciprofloxacin 500 mg twice daily. Intravenous ceftazidime or meropenem was used if piperacillin/tazobactam could not be used because of allergy or antibiotic resistance.

### Procedures

Outpatients were screened based on results from routine microbiological examinations of the lower respiratory tract samples obtained from patients attending the outpatient clinics of the participating respiratory departments. Samples were ordered by clinical staff based on clinical symptoms of exacerbation of the underlying lung disease. Fever, fatigue, peripheral oxygen saturation, and tachypnoea at rest were used as parameters to guide the staff when assessing the need for hospitalisation. Antibiotics were administered in-hospital, since home-treatment with intravenous antibiotics was not available for all study sites at the time of implementation. However, between 2020 and 2022, one site (initiated in 2017), was able to provide treatment at home. We allowed a delay of initiating antibiotic treatment for up to six days in initiating therapy, since there, per the eligibility criteria, was no clinical indication for admission. Baseline measurements were obtained on the calendar date of recruitment (day 1) and follow-up visits were scheduled on day 14, 30, 60, 90, and 365. COPD assessment test (CAT), body mass index (BMI), Medical Research Council (MRC) dyspnoea score, and spirometry were assessed at all visits. Blood samples were drawn at day 1 (baseline) and day 14, and the outpatients underwent a high-resolution CT of the lungs at day 14 assess radiological signs of bronchiectasis at baseline.

### Outcomes

The primary outcome was *time to prednisolone and/or antibiotic requiring exacerbation, in a primary or secondary health care sector, or death from day 20 to day 365 from randomisation*. Death was incorporated in the primary outcome to avoid lead-time bias as the death rate is expected to be high in this population of outpatients with severe pulmonary disease and could thus be incorrectly interpreted as protective of exacerbation. We chose to register events after 20 days from randomisation to avoid misclassifying the study intervention as a fulfilment of the primary outcome. A co-primary outcome of "days alive and out of hospital within 365 days" was degraded to the first secondary outcome by the trial statistician in agreement with the trial leadership (JE and JUSJ) since this outcome would be severely underpowered because of the premature closure of the trial (see " Statistical analysis"). This was done before the database was unblinded to the analysis (see Additional file 1).

The secondary outcomes were: *1) days alive and without hospitalisation from day 20 to day 365 from randomisation, 2) death within 365 days from randomisation, 3) number of admissions with exacerbation within 365 days from randomisation (defined as referral to emergency room or hospitalisation* [9]*), 4) number of days with non-invasive ventilation or invasive ventilation within 90 days from randomisation, 5) microbiological cure at day 90 (defined as P. aeruginosa-negative sputum culture until day 90; no microbiological cure was defined as a P. aeruginosa-positive sputum culture before or at day 90), 6) clinical cure at day 14 (defined as improvement of clinical signs and symptoms related to P. aeruginosa before or on day 14; clinical failure was defined as persistent or worsening of clinical signs and symptoms related to P. aeruginosa before or on day 14), 7) change in CAT score from randomisation to day 90, 8) change in BMI from randomisation to day 90, 9) change in forced expiratory volume in the first second (FEV*_*1*_*) from randomisation to day 90, and 10) decrease of* ≥ *200 ml in FEV*_*1*_* from randomisation to day 365.*

### Statistical analysis

#### Sample size

The sample size was calculated using a group-sequential design, allowing for one interim analysis at half target recruitment, with a power of 80% to avoid type II error at a two-sided 5% significance level. Based on estimates and indicative figures in previous literature, a total of 150 patients (75 patients in each group) were required for the trial (see Additional file 1 for details) [3, 10–12].

#### Analyses

Data were analysed using intention-to-treat (ITT) principles, including all available data, regardless of whether the participant received the intervention. The primary outcome was also analysed using a modified ITT analysis (in study participants who started but did not complete the intervention) and per protocol analysis (in study participants who completed the entire intervention). Completion of the intervention was defined as 14 days of antibiotic treatment in the dual systemic anti-pseudomonal antibiotic study group, and as no anti-pseudomonal treatment within 14 days from randomisation in the control group. Partial completion to intervention was defined as 1–13 days of antibiotic treatment in the antibiotic study group and ≥ 1 day of *P. aeruginosa*-active antibiotic treatment within 14 days from randomisation in the control group.

Data for the primary outcome analyses was analysed using an unadjusted Cox proportional hazard model and results reported as hazard ratio [HR] and 95% confidence limits. A Kaplan–Meier plot was used to describe the process of exacerbations and death in the study groups. A multivariable Cox proportional hazards model adjusting for sex (male vs. female), CAT-score (< 21 vs. ≥ 21) and FEV_1_% predicted (< 50% vs. ≥ 50%) at randomisation was also conducted. Secondary outcomes were compared between the study groups using t-test or Mann–Whitney U test for continuous data and χ^2^-test for nominal data. Analysis of covariance (ANCOVA) was used to model the effect of the intervention on changes in the mean of continuous outcomes, adjusting for the baseline value. All analyses were done using the statistical software SAS (version 9.4) and R (version 3.4.3). Sample size calculation was done using StudySize 2.0 (Frölunda, Sweden).

The interim analysis was planned at half target recruitment (75 patients), with a focus on reporting data on the primary outcome, all-cause mortality at day 365, microbiological cure at day 14 and assessment of study's futility. An independent data and safety monitoring board (DSMB) was appointed to review the trial's safety, efficacy, and progression (see Additional file 1). Due to the slow recruitment rate during the COVID-19 pandemic, the trial's steering committee decided to stop further recruitment in February 2022, when approximately 1/3 of the planned outpatients had been enrolled in the study. This decision was tested with the DSMB, who endorsed it (see Additional file 1). Due to the considerable reduction of the study size, the primary outcome was conducted solely as a "time to event" analysis, and the "days alive and out of hospital" analysis was degraded from a co-primary outcome to the first secondary outcome. As previously mentioned, this decision was made before data was unblinded to the analysis (see " Outcomes"). Data analyses were performed by an analysis team (TWK and AJ), including a trial statistician (TWK), after the final data from the last outpatients last follow-up visit was entered and the database was locked. All analyses were done before breaking of the randomisation code. The study group was presented to the results and unblinded at a scheduled unblinding-meeting after the analyses were performed.

## Results

**Fig. 1:**
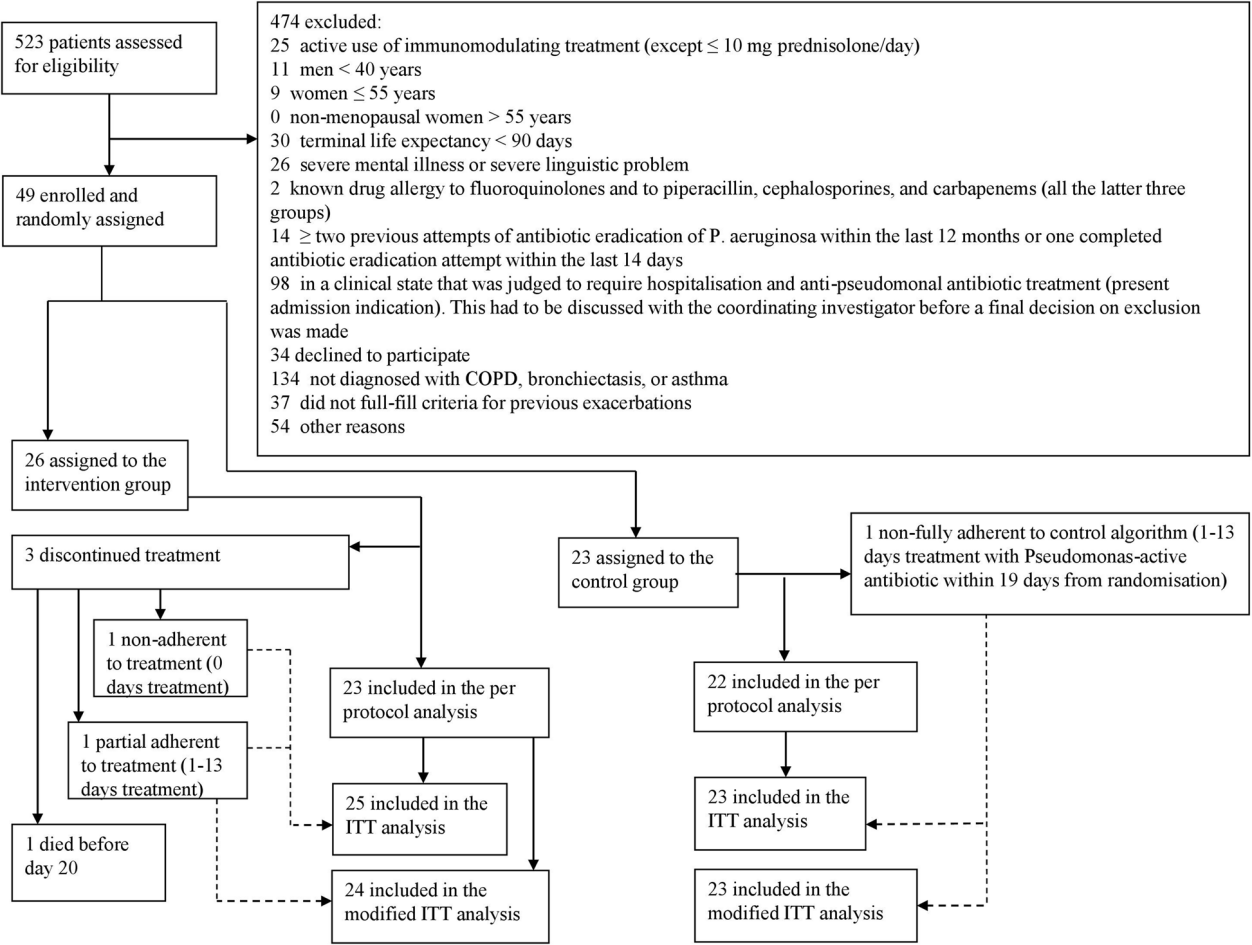
CONSORT flow diagram *ITT* intention-to-treat

A total of 523 outpatients were screened between October 14, 2017, and February 23, 2022. Of these, 49 outpatients (9%) were recruited and randomly assigned to either the antibiotic group (*n* = 26) or the control group (*n* = 23) (Fig. 1). There was complete adherence to the intervention in 92% of the outpatients in the antibiotic group and 96% in the control group. All outpatients in the antibiotic group were treated with combination therapy, except for one case where intravenous monotherapy with meropenem was administered due to antibiotic resistance against ciprofloxacin. Baseline characteristics in the two groups were overall well-balanced regarding demographics, pulmonary function and clinical findings (Table 1). The majority of outpatients had COPD, followed by non-CF bronchiectasis. All but one patient with asthma had a concurrent diagnosis of COPD, or bronchiectasis. A total of 15 (31%) patients were *P. aeruginosa*-naïve prior to the study. All outpatients except one (patient in the antibiotic group who died before day 20) entered the intention-to-treat analysis. There was a 100% follow-up on the primary outcome.

**Table 1:** Baseline characteristics of the intention-to-treat population

	**Antibiotic group (*n* = 26)**	**Control group (*n* = 23)**	**Number missing**	***P*-value**
Age (years), mean (SD)	72 (9)	71 (9)	0	0.80
Sex			0	1.00
Female, n (%)	12 (46)	11 (48)		
Male, n (%)	14 (54)	12 (52)		
Body mass index (kg/m2), median (IQR)	24 (21—28)	26 (21—34)	1	0.30
Smoking			0	0.48
Current, n (%)	1 (4)	2 (9)		
Past, n (%)	20 (77)	19 (83)		
Never, n (%)	5 (19)	2 (9)		
Pack years, median (IQR)	34 (30–40)	42 (30–50)	3	0.14
COPD, n (%)	20 (77)	21 (91)	0	0.25
Bronchiectasis, n (%)	12 (46)	8 (35)	0	0.56
Asthma, n (%)	8 (31)	3 (13)	0	0.18
Exacerbations 12 months prior to inclusion, total, median (IQR)	2 (2—2)	3 (2—5)	0	0.80
**Pulmonary function and symptoms**				
CAT score, mean (SD)	21 (7)	23 (6)	1	0.36
MRC, median (IQR)	3 (2—3)	3 (1—4)	1	0.56
FEV1 (L), median (IQR)	0.94 (0.67—1.25)	0.83 (0.67—1.19)	0	1.00
FEV1 (% predicted), median (IQR)	40 (30—51)	39 (28—52)	0	0.87
FVC (L), median (IQR)	2.07 (1.64—2.69)	1.93 (1.44—2.31)	0	0.61
FVC (% predicted), median (IQR)	69 (64—81)	66 (53—81)	0	0.67
FEV1/FVC ratio (%), median (IQR)	44 (37—58)	51 (36—58)	0	0.54
Home oxygen therapy, n (%)	2 (8)	5 (22)	0	0.23
Home NIV, n (%)	1 (4)	2 (9)	0	0.59
Increased dyspnea, n (%)	10 (38)	16 (70)	0	0.045
Increased cough, n (%)	13 (50)	13 (57)	0	0.78
Increased sputum volume, n (%)	9 (35)	13 (57)	0	0.16
Increased sputum purulence, n (%)	10 (38)	9 (39)	0	1.00
**Comorbidities**				
Ischemic heart disease, n (%)	1 (4)	0 (0)	0	1.00
Heart failure, n (%)	3 (12)	3 (13)	0	1.00
Diabetes, n (%)	0 (0)	6 (26)	0	0.0072
Chronic renal failure, n (%)	1 (4)	0 (0)	0	1.00
Primary immunodeficiency, n (%)	0 (0)	0 (0)	0	1.00
Activities of daily living			0	0.67
Score 0–1, n (%)	22 (85)	21 (91)		
Score 2–4, n (%)	4 (15)	2 (9)		
**Current respiratory medication**				
Long-acting β2 agonist	24 (92)	21 (91)	0	1.00
Long-acting muscarin antagonist	24 (92)	19 (83)	0	0.40
Inhaled corticosteroid	18 (69)	13 (57)	0	0.39
Maintenance oral corticosteroid ≤ 5 mg/day	3 (12)	4 (17)	0	0.69
Maintenance azithromycin	2 (8)	0 (0)	0	0.49
Maintenance inhaled antibiotics	0 (0)	1 (4)	0	0.47
Short-term antibiotics at time of enrolment *	3 (12)	6 (26)	0	0.27
**Clinical findings**				
Systolic blood pressure (mmHg), mean (SD)	141 (23)	139 (16)	1	0.91
Diastolic blood pressure (mmHg), mean (SD)	78 (12)	78 (11)	1	0.57
Heart rate (beats per minute), mean (SD)	81 (12)	90 (13)	1	0.029
Oxygen saturation (%), median (IQR)	94 (92—97)	95 (92—95)	2	0.35
Respiratory rate (breaths per minute), median (IQR)	18 (15—20)	19 (16—20)	1	0.23
Temperature (°C), median (IQR)	36.6 (36.1—37.0)	36.5 (35.8—37.1)	1	0.60

Data are n (%), median (IQR), or mean (SD) unless otherwise specified

*COPD* Chronic obstructive pulmonary disease, *CAT* COPD assessment test, *MRC* Medical research council dyspnea scale, *FEV*_*1*_ Forced expiratory volume the first second, *FVC* Forced expiratory volume, *NIV* Non-invasive ventilation

^*^Antibiotics not active against *Pseudomonas aeruginosa*

### Primary outcome

**Fig. 2:**
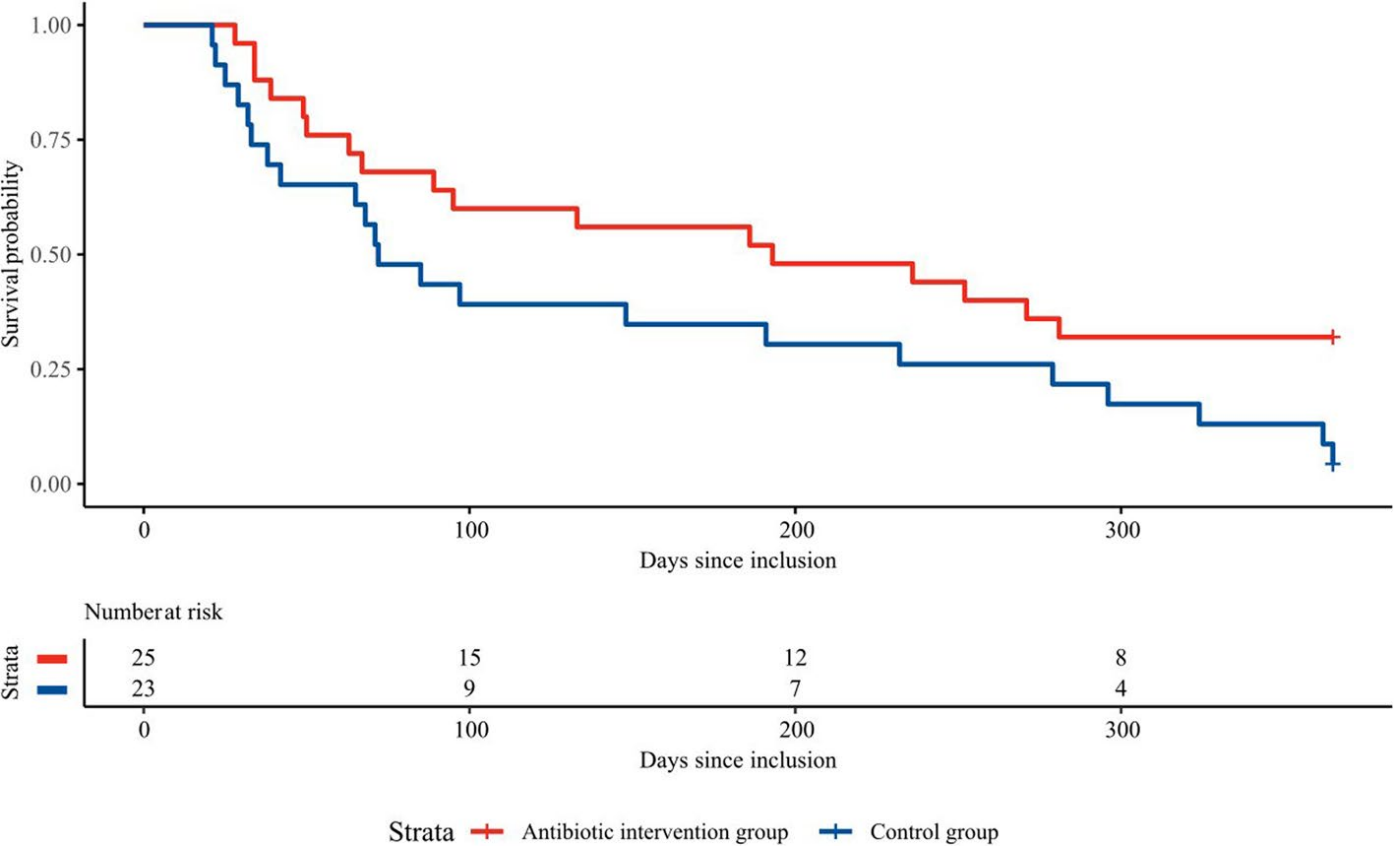
Exacerbation and death from day 20 to 365

*Time to prednisolone or antibiotic requiring exacerbation or death from day 20 to day 365 from randomisation* was increased in the antibiotic group compared to the control group in the intention-to-treat analysis ((HR) 0.51 (95% CI 0.27–0.96), *p* = 0.037), Table 2). Figure 2 illustrates the survival probability using a Kaplan–Meier plot. The result remained stable in the multivariable adjusted Cox proportional hazard regression model, adjusting for sex, CAT-score (< 21 vs. ≥ 21) and FEV1% predicted (< 50% vs. ≥ 50%) at randomisation ((HR) 0.49 (95% CI 0.25–0.95), *p* = 0.034). The signal was unchanged in the modified intention-to-treat analysis but did not reach statistical significance in the per-protocol analysis (Table 2). Due to the small sample size, the lack of statistical significance in the per-protocol analysis might reflect a lack of statistical power, and not a lack of treatment effect of the antibiotic invention. Prior *P. aeruginosa*-status (naïve versus non-naïve) did not alter the signal in the antibiotic group compared to the control group (incident rate ratio: 0.47 in naïve patients versus 0.13 in non-naïve patients). The results remained statistically significant in the population with COPD in a post-hoc analysis differentiating patients by the type of lung disease (Table 1 in Additional file 1).

**Table 2:** Primary outcome measurement

	**Antibiotic group (*n* = 26)**	**Control group (*n* = 23)**			
**Exacerbation or death within days 20 to 365**	**N events (%)**	**N events (%)**	**Crude HR (95% CI)**	***P* value**	**Not included in analysis**
ITT*	17 (68)	22 (96)	0·51 (0.27—0.96)	0.037	1
Per protocol	16 (70)	21 (95)	0.55 (0.29—1.06)	0.072	4
Modified ITT	16 (67)	22 (96)	0.49 (0.26—0.94)	0.032	2

Data are mean (95% CI) or n (%) unless otherwise specified

*ITT* Intention-to-treat, *HR* Hazard ratio

^*^One participant was excluded from this analysis as this participant died before day 20 and the primary outcome was defined as having to occur day 20–365

### Secondary outcomes

Numerically, the *days alive and without hospitalisation from day 20 to day 365* was higher in the dual systemic anti-pseudomonal antibiotic group, although this did not reach statistical significance (mean 315 days (95% CI 281–348) in the antibiotic group vs. 288 days (95% CI 246–329) in the control group, *p* = 0.31; Table 3 and Fig. 3 in Additional file 1). *Death within 365 days from randomisation* occurred in two outpatients (8%) in the dual-systemic anti-pseudomonal antibiotic group and five outpatients (22%) in the control group (*p* = 0.23; Table 3 and Fig. 4 in Additional file 1).

**Table 3:** Secondary outcomes measurements

	**Antibiotic group (*n* = 26)**	**Control group (*n* = 23)**	***P* value**	**Number missing**
Days alive and out of hospital from day 20 to day 365				0
Parametric analysis, mean (95% CI)	315 (281—348)	288 (246—329)	0.31	
Non-parametric analysis, median (IQR)	343 (336—345)	325 (296—345)	0.31	
Death within 365 days, n (%)	2 (8)	5 (22)	0.23	0
Microbial cure at l day 90, n (%)	11 (42)	5 (22)	0.14	0
Clinical cure at day 14, n (%)	18 (78)	8 (38)	0.013	5
Decrease ≥ 200 mL FEV1 from day 0 to 90, n (%)	0 (0)	0 (0)	1.00	6
Admissions with exacerbations within 365 days, mean (95% CI)	1.1 (0.6—1.7)	2.9 (1.3—4.5)	0.037	2
Days on NIV or mechanical ventilation within 90 days, mean (95% CI)	0.04 (-0.04—0.12)	0.13 (-0.02—0.28)	0.27	0
Change in CAT from baseline to 90, mean (95% CI)	-3.9 (-6.3—-1.5)	-2.7 (-5.1—0.2)	0.33	6
Change in BMI from baseline to day 90, mean (95% CI)	-0.3 (-0.7—0.2)	-0.9 (-1.8—0.0)	0.36	6
Change in FEV1 from baseline to day 90, mean (95% CI)	0.07 (-0.03—0.18)	0.01 (-0.04—0.05)	0.20	6

Data are mean (95% CI) or n (%) unless otherwise specified

*COPD* Chronic obstructive pulmonary disease, *NIV* Non-invasive ventilation, *CAT* COPD assessment test, *BMI* Body mass index, *FEV*_*1*_ Forced expiratory volume the first second

The *number of admissions with exacerbation within 365 days from randomisation* was different: mean 1.1 (95% CI 0.6–1.7) in the antibiotic group versus 2.9 (95% CI 1.3–4.5) in the control group, *p* = 0.037, Table 3 and Fig. 5 in Additional file 1. Further, *clinical cure at day 14* was 78% in the antibiotic group versus 38% in the control group, *p* = 0.013; Table 3.

There was no difference between the two study groups in the remaining secondary outcomes (Table 3); *The mean number of days with non-invasive ventilation or invasive ventilation within 90 days from randomisation* was 0.04 (95% CI -0.04–0.12) in the antibiotic group compared to 0.13 (95% CI -0.02–0.28) in the control group (*p* = 0.27), and the occurrence of *microbiological cure at day 90* was 42% of the outpatients in the antibiotic group compared to 22% in the control group (*p* = 0.14). The number of patients with *P. aeruginosa*-positive sputum samples according to each study visit are displayed in Table 2 in Additional file 1. We did not detect any significant *change in CAT scores, BMI, or FEV*_*1*_* from randomisation to day 90, nor a decrease of* ≥ *200 ml in FEV*_*1*_* from randomisation to day 365.* However, as illustrated in Fig. 6 in Additional file 1, there was an apparent trend towards decreased CAT score, increased BMI, and increased FEV_1_ from randomisation to day 30 in the antibiotic group. The association was explored in a post-hoc analysis and was statistically significant for FEV_1_ (Table 3 in Additional file 1). Moreover, there were few adverse effects, and no severe adverse effect associated to the intervention, in the study population (Table 4 in Additional file 1). Due to the slow progression in recruiting patients during the COVID-19 pandemic lockdown, the trial was stopped prematurely in February 2022 (see "Methods").

## Discussion

We conducted a multicenter, randomised, controlled, open-label trial to evaluate the efficacy of dual systemic anti-pseudomonal antibiotics in outpatients with pre-existing lung disease and a recent respiratory tract culture sample with *P. aeruginosa.* We found that the risk of prednisolone or antibiotic requiring exacerbation or death within one year was reduced to about half. The total number of hospitalisation-requiring exacerbations within one year was reduced from almost three to approximately one, and clinical cure at day 14 also improved markedly. In all other secondary outcomes, we observed a non-significant trend in the direction of benefit from the dual systemic antibiotic intervention. These included: *i)* days alive and without hospitalisation from day 20 to 365 from randomisation, *ii)* death from all causes within 365 days, *iii)* number of days with non-invasive ventilation or invasive ventilation within 90 days, *iv) *microbiological cure at day 90, *v) *change in CAT score to day 90, *vi)* change in BMI to day 90, *vii)* change in FEV_1_ to day 90, and *viii)* decrease of ≥ 200 ml in FEV_1_ from randomisation to day 365. No secondary outcomes trended towards harm from the intervention. The majority of outpatients had COPD, followed by non-CF bronchiectasis. The main result seemed to be preserved both among *P. aeruginosa*-naïve and *P. aeruginosa*-non-naïve patients.

To our knowledge, this is the first randomised controlled trial to explore the clinical effects of systemic antibiotic treatment targeting *P. aeruginosa* in patients with pre-existing lung disease and frequent exacerbations. Previously, a smaller and retrospective observational study conducted between 2004–2010 assessed the effects of different antibiotic regimens after the first colonisation of *P. aeruginosa*in 30 patients with non-CF bronchiectasis, in whom the majority were treated with systemic antibiotics for two weeks. Exacerbation frequencies seemed lower after antibiotic treatment [6].

In the past two decades, a growing number of trials have investigated the potential clinical advantages of inhaled antibiotic as eradication treatment in patients with lung disease and recurrent isolation of *P. aeruginosa*. Recent meta-analyses have highlighted some controversy regarding their impact on exacerbations in non-CF bronchiectasis [13, 14]. To date, no randomised controlled trials have tested inhaled antibiotics in patients with asthma or COPD.

Suggestions for combination treatment for *P. aeruginosa* are based on in vitro data [15] and observational studies [8]. Due to important limitations concerning study design and sample size, no clear evidence of the benefits of combination therapy over monotherapy for *P. aeruginosa*bacteraemia has been proposed [16, 17].

Our study was stopped prematurely based on the collapse of recruitment during the COVID-19 pandemic. This decision was made by the trial Steering Committee without any knowledge of the data. The appointed Data and Safety Monitoring Board endorsed the decision. The premature halt of the trial is disappointing, however, our data remains the only trial data on this important question, and further, they are supported by observational studies and microbiological evidence. They do, in fact, inform on a clinical subject to which clinical practice is highly differing worldwide. The signal of the results seems very strong, and importantly, is consistent between the primary outcome and secondary outcomes, and there was 100% follow-up on all outcomes.

Our study's relatively low bacterial eradication rate is in line with findings in previous trials of combined inhaled and systemic antibiotic treatment in non-CF bronchiectasis [5, 18]. Our observations are not surprising as *P. aeruginosa*is known to grow persistently in the airways of patients with chronic pulmonary diseases [3, 19–24]. This was also demonstrated in a sub-study of the present trial, in which we conducted a whole-genome-sequencing on the systematically collected sputum samples in the initial 23 outpatients. This analysis revealed that subsequent growth of *P. aeruginosa* was common, with 83% experiencing it during the 365-day follow-up period. Furthermore, the recurrent *P. aeruginosa*-positive sputum samples harbour the same *P. aeruginosa*clone as the first sputum culture at recruitment [25]. Thus, using dual systemic anti-pseudomonal antibiotic therapy is unlikely to provide a sufficient long-term eradication in patients with pre-existing lung disease. Consequently, the term *eradication therapy* used for dual systemic antibiotics, should be avoided. However, there seems to be possible important clinical short-term effects in terms of a higher clinical cure rate as well as patient reported symptoms (CAT score) and improvement in FEV_1_following antibiotic treatment. To our best knowledge, similar improvements in pulmonary function have not been observed in any previous trials assessing inhaled anti-pseudomonal antibiotics in non-CF bronchiectasis [14, 15].

Our trial has limitations apart from the premature closure. Second, we used an unblinded intervention. Prior to study start, the trial steering committee discussed the possibility for double blinding, but the investigators from the sites found it highly unfeasible to convince the department heads to allow admission for until 14 days, just to receive placebo. Thus, despite the multicenter and randomised study design, this could have affected the assessment of some outcomes, including clinical cure assessment, which was symptom-based and not quantified.

In conclusion, dual systemic antibiotic treatment against *P. aeruginosa* markedly improved critical clinical outcomes like exacerbations in outpatients with COPD, non-CF bronchiectasis or asthma with no clinical reason for admission. Our study thus demonstrates, the severe limitation of premature closure held in mind, that dual antibiotics for two weeks in outpatients with COPD, non-CF bronchiectasis or asthma who have a culture sample with *P. aeruginosa,* and who are not judged as in clinical need of hospitalisation, is well-tolerated and leads to substantially better clinical outcomes within one year. Such an intervention should be considered in patients like the ones included in our trial.

### Supplementary Information

Additional file 1: 1. Trial group. 2. Study timeline. 3. Study protocol. 4. Statistical analysis plan. 5. Randomisation and masking. 6. Adherence to treatment. 7. Protocol amendment log. 8. Data and Safety Monitoring Board (DSMB) charter. 9. Patient recruitment by trials site. 10. DSMB endorsement letter for early trial termination. 11. Note to statistical analysis plan. 12. Supplemental tables and figures.

## Data Availability

Requests for data, including a study protocol, should be sent to the principal investigator, who will review the request with the TARGET ABC steering committee. If the hypothesis of the request is within the informed consent granted by study participants at the time of inclusion, and the hypothesis is judged to be valid, a data transfer agreement will be prepared in agreement with national legislation for data sharing.

## Abbreviations

BMI: Body mass index
CI: Confidence interval
COPD: Chronic obstructive pulmonary disease
COVID-19: Coronavirus disease 2019
CT: Computer tomography
DSMB: Data and safety monitoring board
FEV1: Forced expiratory volume the first second
GCP: Good clinical practice
HR: Hazard ratio
MRC: Medical research council
NIV: Non-invasive ventilation
Non-CF: Non-cystic fibrosis
ITT: Intention-to-treat
