# Supplementary material for: Systemic antibiotics for Pseudomonas aeruginosa infection in outpatients with non-hospitalised exacerbations of pre-existing lung diseases: a randomised clinical trial
Source: Respir Res. 2024 Jun 6;25:236. doi: 10.1186/s12931-024-02860-9 (PMC11157704; doi:10.1186/s12931-024-02860-9)

Additional File:

TARGETed AntiBiotiCs (TARGET-ABC) multicenter, randomized, controlled, open-label trial

**Authors:**

Josefin Eklöf MD, Imane Achir Alispahic, Karin Armbruster, Therese Sophie Lapperre, Andrea Browatzki, Rikke Holmen Overgaard, Zitta Barrella Harboe, Julie Janner, Mia Moberg, Charlotte Suppli Ulrik, Helle Frost Andreassen, Ulla Møller Weinreich, Jakob Lyngby Kjærgaard, Jenny Villadsen, Camilla Sund Fenlev, Torben Tranborg Jensen, Christina Wellendorph Christensen, Jette Bangsborg, Christian Ostergaard, Khaled Saoud Ali Ghathian, Alexander Jordan, Tobias Wirenfeldt Klausen, Thyge Lynghøj Nielsen, Torgny Wilcke, Niels Seersholm, Pradeesh Sivapalan, Jens-Ulrik Stæhr Jensen.

[THE TARGET-ABC TRIAL GROUP 3](#_Toc149311661)

[Study director 3](#_Toc149311662)

[Steering Committee: 3](#_Toc149311663)

[Data Safety and Monitoring Board: 3](#_Toc149311664)

[GCP unit — Research: 3](#_Toc149311665)

[Trial statisticians: 3](#_Toc149311666)

[Trial site investigators and research staff 3](#_Toc149311667)

[STUDY TIMELINE 4](#_Toc149311668)

[STUDY PROTOCOL 5](#_Toc149311669)

[1. Background 5](#_Toc149311670)

[2. Aim and hypothesis 6](#_Toc149311671)

[3. Method 6](#_Toc149311672)

[3.1 Design 6](#_Toc149311673)

[3.2 Randomisation 6](#_Toc149311674)

[3.3 Enrolment and Inclusion 6](#_Toc149311675)

[3.4 Inclusion criteria: 7](#_Toc149311676)

[3.5 Exclusion criteria: 7](#_Toc149311677)

[4. Data collection 7](#_Toc149311678)

[5. Research biobank 8](#_Toc149311679)

[6. Statistical considerations and power sample calculation 8](#_Toc149311680)

[6.1 Primary outcome 10](#_Toc149311681)

[6.2 Secondary outcomes 10](#_Toc149311682)

[7. Adverse effects and risks 10](#_Toc149311683)

[8. Removal from and interruption of the trial 11](#_Toc149311684)

[9. Funding 11](#_Toc149311685)

[10. Access to data 11](#_Toc149311686)

[11. Publication of study results 11](#_Toc149311687)

[12. Scientific ethical statement 11](#_Toc149311688)

[13. Protocol references 11](#_Toc149311689)

[STATISTICAL ANALYSIS PLAN 13](#_Toc149311690)

[RANDOMISATION AND MASKING 18](#_Toc149311691)

[ADHERENCE TO TREATMENT 18](#_Toc149311692)

[PROTOCOL AMENDMENT LOG 18](#_Toc149311693)

[DATA AND SAFETY MONITORING BOARD CHARTER 19](#_Toc149311694)

[PATIENT RECRUITMENT PER TRIAL SITE 23](#_Toc149311695)

[DSMB ENDORSMENT LETTER FOR EARLY TERMINATION 24](#_Toc149311696)

[NOTE TO STATISTICAL ANALYSIS PLAN PRIOR TO DATABASE UNLOCKING 25](#_Toc149311697)

[SUPPLEMENTAL TABLES AND FIGURES 26](#_Toc149311698)

# THE TARGET-ABC TRIAL GROUP

Study director**:** Jens Ulrik Stæhr Jensen, Herlev and Gentofte University Hospital
Principal investigator: Josefin Eklöf, Herlev and Gentofte University Hospital

Steering Committee: Jens-Ulrik Stæhr Jensen, Jørgen Vestbo, Julie Janner, Mia Moberg, Therese Lappere, Charlotte Suppli Ulrik, Torgny Wilcke, Kristoffer Marså, Pia Thaning, Helle Frost Andreassen, Ulla Møller Weinreich, Andrea Browatzki, Uffe Bødtger, Ingrid Titlestad, Elisabeth Bendstrup Christian B. Laursen Thomas Benfield, Thomas Benfield Christian Meyer and Merete Raaschou.

Data Safety and Monitoring Board: Tobias Wirenfeldt Klausen, Tor Biering-Sørensen, Philipp Schuetz

GCP unit — Research: Lotte Lund, Sanne Andersen, Charlotte Calov

Trial statisticians: Tobias Wirenfeldt Klausen, Alexander Jordan

## Trial site investigators and research staff

*Herlev and Gentofte University Hospital:* Karin Armbruster, Niels Seersholm, Jon Torgny Wilcke, Pradeesh Sivapalan, Imane Achir Alispahic, Rikke Holmen Overgaard, Jenny Villadsen, Jakob Lyngby Kjærgaard, Christina Wellendorph Christensen, Jette Bangsborg.

*Bispebjerg University Hospital:* Therese Sofie Lapperre, Helle Frost Andreassen

*Hospital of Southwest Jutland:* Torben Tranborg Jensen, Tilde Ellingsgaard

*Odense University Hospital:* Sofie Lock Johansson

*North Zealand University Hospital:* Rikke Holmen Overgaard, Camilla Fenlev, Andrea Browatzki, Zitta Barrella Harboe, Thyge Lynghøj Nielsen

*Amager and Hvidovre University Hospital:* Julie Janner, Mia Moberg, Marie Heidemann, Charlotte Suppli Ulrik, Christian Ostergaard, Khaled Saoud Ali Ghathian,

*Aalborg University Hospital:* Ulla Møller Weinreich, Roxanna Vijdea

# STUDY TIMELINE


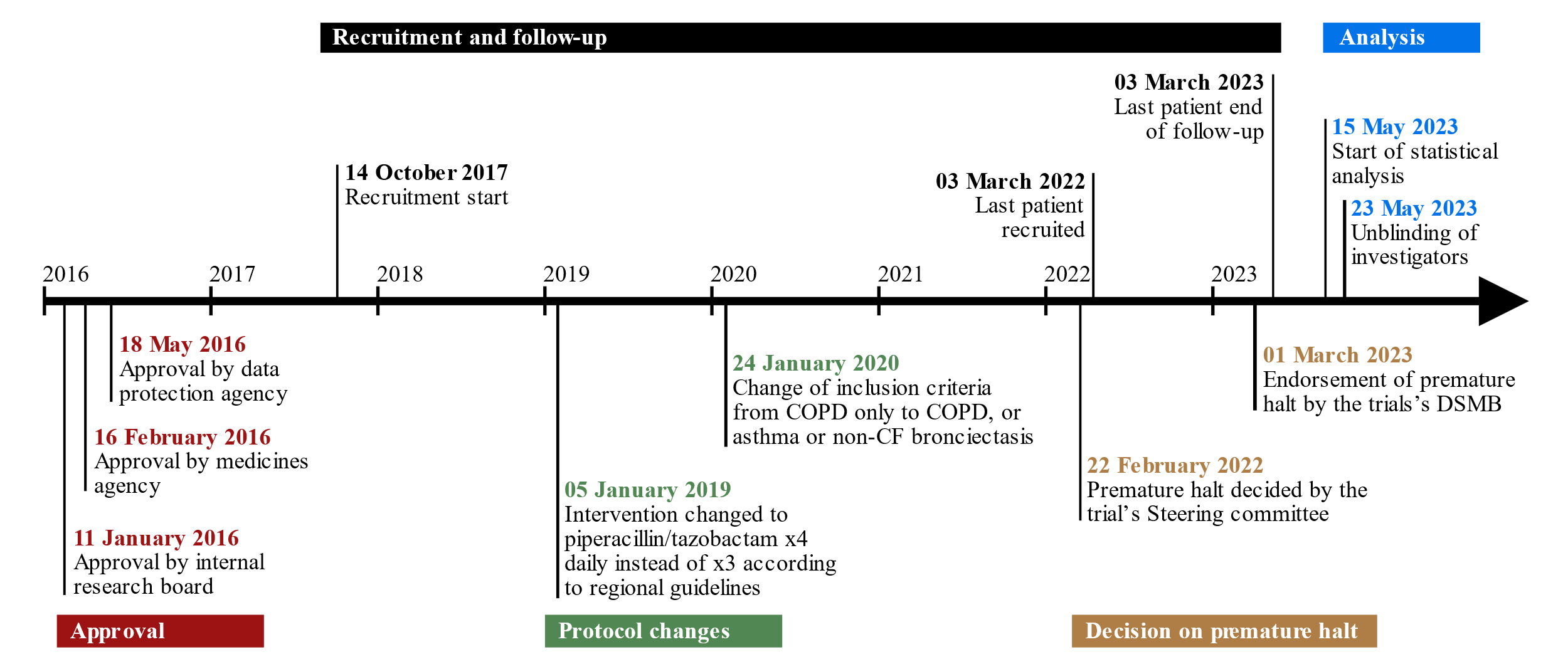


# STUDY PROTOCOL

**Targeted AntiBiotics for Chronic pulmonary diseases (TARGET-ABC):**

**Can targeted antibiotic therapy improve the prognosis of *Pseudomonas aeruginosa* infected patients with chronic pulmonary obstructive disease, non-cystic fibrosis bronchiectasis or asthma?**

**A multicenter, randomised, controlled, open-label trial**

**Scientific Project Sponsor**

Chronic Obstructive Pulmonary Disease Trial Network (COP:TRIN) - a network of independent COPD research in Denmark

Chair: Jens-Ulrik Stæhr Jensen, MD, PhD, Associate Professor

**Coordinator Investigator**

Josefin Eklöf, MD, PhD student, Department of Internal Medicine, Section of Respiratory Medicine, Herlev and Gentofte University Hospital, Copenhagen, Denmark

**Principal Supervisor**

Jens-Ulrik Stæhr Jensen, MD, PhD, Associate Professor Sponsor, Department of Internal Medicine, Section of Respiratory Medicine, Herlev and Gentofte University Hospital, Copenhagen, Denmark

**Co-supervisors**

Torgny Wilcke, MD, PhD, Associate Professor, Department of Internal Medicine, Section of Respiratory Medicine, Herlev and Gentofte University Hospital, Copenhagen, Denmark

Niels Seersholm, MD, DMSc, Department of Internal Medicine, Section of Respiratory Medicine, Herlev and Gentofte University Hospital, Copenhagen, Denmark

## 1. Background

COPD, non-CF bronchiectasis, and asthma are common chronic pulmonary diseases and important causes of death and disability worldwide [1,2,3]. These diseases are characterized by shared common symptoms such as productive cough and susceptibility to recurrent exacerbations that are often associated with infections. These exacerbations lead to accelerated loss of lung function, reduced quality of life, and increased morbidity and mortality and have major socio-economic consequences [4,5].

Compared to COPD and asthma, which both are diagnosed on the basis of airflow obstruction and therefore are physiological diagnoses, bronchiectasis is a structural diagnosis with the presence of permanent airway dilatation on radiological imaging [4,5,6]. However, the co-existence of bronchiectasis and asthma or COPD is common [7].

*Pseudomonas aeruginosa* [8] has been reported to be present in the lower airways in up to 20% of patients with COPD [9,10,11] and is frequently detected in patients with non-CF bronchiectasis [12]. The bacterium has also been observed in patients with asthma [13]. Nevertheless, the influence of *P. aeruginosa* on the progression of these diseases is far from fully elucidated. The bacterium is seen primarily in advanced diseases with severely impaired lung function [12,13,14] and is associated with increased frequency of exacerbation, prolonged hospitalization, and poor long-term prognosis with increased mortality rates compared to *P. aeruginosa*-negative patients [15].

However, since an impairment of lung function itself is a strong predictor of morbidity and mortality, it is not certain whether infection with *P. aeruginosa* is secondary to lung function impairment or whether the presence of *P. aeruginosa* itself leads to pulmonary tissue inflammation and remodeling, impaired lung function, and overall poor prognosis.

Thus, the role of *P. aeruginosa* on the progression of COPD, non-CF bronchiectasis, and asthma is poorly characterized. To date, evidence-based guidelines for the management and treatment of *P. aeruginosa* infection are lacking, and the management of *P. aeruginosa* is often based on expert consensus and studies of other chronic lung diseases, including CF. In CF, *P. aeruginosa* is a leading cause of morbidity and early death with evidence of improved clinical outcomes through aggressive and targeted antibiotic treatment [16]. In Denmark, the first treatment choice for clinically treatment-requiring *P. aeruginosa* infection is usually 10–14 days of antibiotic combination therapy with intravenous piperacillin/tazobactam and oral ciprofloxacin [17].

With this randomized controlled trial, we aim to increase the understanding of the clinical significance and consequences of *P. aeruginosa* infection in patients with chronic, non-CF pulmonary disease. The main purpose is to investigate if targeted, antibiotic treatment of *P. aeruginosa* improves the disease prognosis in patients with exacerbation of COPD, non-CF bronchiectasis or asthma, and *P. aeruginosa*-positive lower respiratory tract culture sample.

## 2. Aim and hypothesis

The aim of this study is to investigate whether antibiotic treatment for *P. aeruginosa* can improve prognosis in patients with COPD, non-CF bronchiectasis or asthma. Our hypothesis is, that antipseudomonal antibiotics improve prognosis by reducing exacerbations.

## 3. Method

## 3.1 Design

The study is a multicenter, randomized, controlled, open-label trial in outpatients with COPD, non-CF bronchiectasis, and asthma with current *P. aeruginosa*-positive lower respiratory tract sample. Study participants are followed for 1 year. Participants are recruited by investigators who are employed at the participating respiratory outpatient clinics in Denmark.

Patients will be randomised 1:1 to one of the two study groups:

1. Intervention group: antibiotic treatment (administered in-hospital)
2. Control group: no antibiotic treatment

The first choice of antibiotic treatment is dual therapy with intravenous piperacillin/tazobactam 4 /0,5 gram 4 times daily and oral ciprofloxacin 500 mg 2 times daily for 14 days. In case of penicillin allergy or antibiotic resistance, intravenous piperacillin/tazobactam is replaced by intravenous ceftazidime or meropenem. In case of fluoroquinolone allergy or antibiotic resistance, intravenous beta-lactam is given as monotherapy.

##

## 3.2 Randomisation

Randomisation is conducted using a secure web application (REDCap; www.projectredcap.org) where inclusion and exclusion criteria are required to be filled out correctly in order to randomise a study participant. Pre-stratified block randomisation with blocks of varying and blinded size is applied to ensure equal distribution of patients in the study groups based on study center (respiratory outpatient clinic) and age (above or below 70 years of age).

## 3.3 Enrolment and Inclusion

All consecutive outpatients with COPD, non-CF bronchiectasis, or asthma and current *P. aeruginosa*-positive respiratory sample from the participating study centres (respiratory departments) are considered for study enrollment. Patients are invited to participate in the trial if they fulfil the following inclusion and exclusion:

## 3.4 Inclusion criteria:

- *P. aeruginosa*-positive lower respiratory tract sample
- COPD, non-CF bronchiectasis, or asthma verified by a respiratory specialist based on clinical assessment and additional tests:

1. COPD: spirometry

b) Asthma: reversibility

c) Non-CF bronchiectasis: high-resolution computed tomography scan

- Minimum of two previous exacerbations, or one previous hospitalization-requiring or emergency room-demanding exacerbation, with the treatment of systemic prednisolone and/or antibiotics within the last 12 months
- Written informed consent

## 3.5 Exclusion criteria:

- Immunomodulating treatment (except ≤ 10 mg prednisolone/day)
- Men < 40 years
- Women ≤ 55 years
- Non-menopausal women > 55 years (i.e., menstruation within the last 12 months)
- Life expectancy < 90 days
- Severe mental illness or severe linguistic problem
- Known drug allergy to (i) fluroquinolone and (ii) both penicillin/piperacillin, cephalosporin, and carbapenems
- ≥ 2 previous eradication attempts of *P. aeruginosa* within the last 12 months or 1 completed within the last 14 days *
- Patients who clinically require hospitalization and anti-pseudomonal antibiotic treatment. This exclusion criterion must be discussed with the coordinating investigator before the final decision on exclusion is made

*Defined as 10-14 days of dual therapy with beta-lactam and fluoroquinolone.

## 4. Data collection

The daily project management is carried out by the primary investigators and sub-investigators, consisting of health professionals from the departments involved in the trial, and is coordinated by the coordinating investigator (Josefin Eklöf). Data is collected in electronical case report forms in Redcap, specific to each participant., and include demographic data, health status, hospitalisations, clinical parameters, study results and prescribed medication are recorded. Case report forms are archived for 15 years. Collection and storage of data is in compliance with Good Clinical Practice (GCP) guidelines and is regularly monitored by local GCP units.

Study overview is summarised in Table 1. Follow-up visits are scheduled after 14, 30, 60, 90 and 365 days. Blood samples are taken at the start of the study and before administration of any antibiotics, after 14 days and continuously every 3 days during antibiotic treatment. Antibiotic therapy is started according to the current guidelines for each preparation, incl. adjustment regarding the participant’s usual medication, kidney function, age, side effects and possibly allergies. Administration of antibiotics is registered and recorded using the department's applicable electronic medicine module. HRCT (high-resolution computed tomography) is performed at the start of the study in order to identify underlying prevalence of emphysema and bronchiectasis.

**Table 1.** Study overview

| **Study period** | | | | | | | | |
| --- | --- | --- | --- | --- | --- | --- | --- | --- |
|  |  | **Enrolment** | **Intervention** | **Follow-up** | | | | |
| **Visit number** | | **1** |  | **2** | **3** | **4** | **5** | **6** |
| **Study day** | | **0** | **0-14** | **14** | **30** | **60** | **90** | **365** |
|  | |  |  |  |  |  |  |  |
| **Enrolment** | |  |  |  |  |  |  |  |
| Eligibility screening | | X |  |  |  |  |  |  |
| Informed consent | | X |  |  |  |  |  |  |
| Randomisation | | X |  |  |  |  |  |  |
| **Study arm** | |  |  |  |  |  |  |  |
| Intervention group: antibiotic treatment, in-hospital | |  | X |  |  |  |  |  |
| Control group: no antibiotic treatment, not hospitalised | |  |  |  |  |  |  |  |
| **Data collection and examinations** | |  |  |  |  |  |  |  |
| Demographics | | X |  | X |  |  |  |  |
| Sputum sample | | X |  | X | X | X | X | X |
| Body mass index (BMI) | | X |  | X | X | X | X | X |
| Medical Research Council Dyspnoea Scale (MRC) | | X |  | X | X | X | X | X |
| COPD Assessment Test (CAT) | | X |  | X | X | X | X | X |
| Spirometry | | X |  | X | X | X | X | X |
| Vital parameters | | X |  | X |  |  |  |  |
| High resolution computed tomography (HRCT) | | X |  |  |  |  |  |  |
| Blood samples | | X | X | X |  |  |  |  |

## 5. Research biobank

A research biobank with blood and sputum samples collected at enrolment and follow-up visits have been established. This biobank will be used for future research, including genomic analyses of *P. aeruginosa*-positive sputum samples collected during the study. Separate information and consent material for the study participants have been prepared and the Danish Science Ethics Committee and the Danish Data Protection Agency. The samples are stored in an anonymously form for 15 years. The material is reserved for the current research study and can only be used for other research projects with the permission from the Danish Science Ethics Committee.

## 6. Statistical considerations and power sample calculation

Data will be analyzed using intention-to-treat (ITT) principles, including all the data available, regardless of whether the intervention was completed or not. The aim of the ITT analysis is also to provide unbiased comparisons among the two study groups and to avoid the effects of potential study dropouts and protocol deviations.

Patients in the control group will be compared to patients in the intervention group. We will use Fisher’s exact test and chi-squared test for dichotomous outcomes and *T*-test for continuous outcomes. The timed dichotomous outcomes will be visualized through Kaplan-Meier plots. Furthermore, adjusted analyses will be performed with a multivariable Cox proportional hazards model, adjusting for baseline variables and calculating hazard ratios.

Data will be processed and analyzed in SAS and graphs are generated in Microsoft Excel and other graph programs.

The sample size is calculated based on 80% power, a two-sided 5% significance level and the following estimates and indicative figures for COPD:

1. *P. aeruginosa* incidence 5-20%
2. 67% of study participants in the antibiotic-free group have exacerbated or died within 12 months
3. 47% of study participants in the antibiotic group have exacerbated or died within 12 months

Thus, we expect an effect size of 20% absolute reduction (30% relative reduction) of exacerbation or mortality in the antibiotic group. Based on the above, a total of 150 patients should be included. Furthermore, to avoid error estimates and risk of including too few patients (underpowering), the study is "event driven" and will only be closed when at least 67% of patients in the antibiotic-free group have exacerbated or died, but twelve months must have passed, also if more than 67% of patients have experienced the primary outcome event.

For non-CF bronchiectasis and asthma:

1. Annual exacerbation rate with *P. aeruginosa*: 2.85
2. Annual exacerbation rate without *P. aeruginosa*: 1.80
3. Standard deviation, annual exacerbation rate: 1.5

Based on these reference estimates [18], a total of 66 patients should be included (i.e., 33 patients in each study group). Since most study participants potentially could be COPD patients, the number from the COPD sample size calculation is used in order not to risk underpowering the study. If non-COPD patients are recruited, the power will thus be increased to > 80%.

In cases of low *P. aeruginosa* incidence (PAi), patients will need to be recruited from several pulmonary departments to achieve the desired sample size, as calculated below. The figures are based on estimation of approximately 2,000 patients with GOLD Class C or D/outpatient clinic. Of these, 1/3 are expected to be able to produce sputum sample. However, the dropout rate is estimated to be about the same level, thus there are approximately potentially 400 patients/outpatient clinic/year.

5% PAi: 0.05 x 400 = 20 patients/outpatient clinic/year x 8 outpatient clinics = 160 patients / year

10% PAi: 0.10 x 400 = 40 patients/outpatient clinic/year x 4 outpatient clinics= 160 patients/year

15% PAi: 0.15 x 400 = 60 patients/outpatient clinic/year x 3 outpatient clinics= 180 patients/year

20% PAi: 0.20 x 400 = 80 patients/outpatient clinic/year x 2 outpatient clinics= 160 patients/year

An independent Data Safety Monitoring Board will monitor the safety of the trial by conducting interim analyses based on primary and secondary endpoints when half of the study population (i.e. 75 patients) has completed the study.

## 6.1 Primary outcome

- Time to prednisolone and/or antibiotic requiring exacerbation, in primary or secondary health care sector, or death from day 20 to day 365 from randomisation *

## 6.2 Secondary outcomes

- Days alive and without hospitalisation from day 20 to day 365 from randomisation
- Death within 365 days from randomisation
- Number of admissions with exacerbation of the chronic lung disease within 365 days from randomisation
- Number of days with non-invasive-ventilation or invasive ventilation within 90 days from randomisation
- Microbiological cure **
- Clinical cure ***
- Change in COPD Assessment Test (CAT) between baseline and day 90 from randomisation
- Change in BMI between baseline and day 90 from randomisation
- Change in FEV_1_ between baseline and day 90 from randomisation
- Decrease of ≥ 200 ml in FEV_1_ from randomisation to day 365

*The co-primary outcome of "days alive and without hospitalisation from day 20 to day 365 from randomisation" was degraded to the first secondary outcome by the trial statistician in agreement with the coordination investigator and study director since this outcome would be severely underpowered because of the premature closure of the trial. This decision was taken before the database was unblinded to the analysis (see Supplementary page 24).

**Microbiological cure: *P. aeruginosa*-negative sputum culture until day 90. No microbiological cure: positive sputum culture with clonally same *P. aeruginosa* strain ≤ day 90. Re-infection: positive sputum sample with non-clonally same *P. aeruginosa* strain ≤ day 90.

***Clinical cure: cessation or improvement of clinical signs and symptoms related to *P. aeruginosa* ≤ day 14. Clinical failure: persistent or worsening of clinical signs and symptoms related to *P. aeruginosa* ≤ day 14.

## 7. Adverse effects and risks

Treatment with piperacillin/tazobactam and ciprofloxacin are associated with a low frequency of serious side effects (<0.01%). These include pancytopenia, bone marrow depression, psychological reactions and depression, seizures, anaphylactic reaction/shock, renal failure, Steven-Johnson syndrome, toxic epidermal necrolysis, liver necrosis, ventricular arrhythmia and pseudomembranous colitis. The Summary of Product Characteristics (SCP) will be used as a reference document when assessing adverse reactions. As the study is focusing on the long-term clinical effects of the drugs, only unknown adverse reactions not recorded in the SPC will be registered in the trial.

The following events are expected and naturally occurring as part of the underlying lung disease and lung infection and do not need be recorded as adverse events: coughing, respiratory mucus, shortness of breath, wheezing, chest pressure, palpitations, lower leg swelling, unrest, anxiety, sleep disorders, eating disorders, fatigue, dizziness and abnormalities in blood test responses that are related to infection and that are not considered clinically significant. Likewise, exacerbation of COPD, non-CF bronchiectasis or asthma will not be registered as a serious adverse event as occurrence of this is already included as (primary) endpoint, and thus will be assessed by the DSMB in the pre-planned interim analysis. All other serious adverse events or reactions must be immediately reported (= within 24 hours of investigator becoming aware of a serious adverse event or reaction) to the sponsor to assess if a serious related adverse event is unexpected and thus possibly a suspected unexpected serious adverse reactions (SUSAR). All registered adverse events and adverse reactions will be reported at the end of the trial in a final report to the Danish Health Authority. All serious adverse reactions and adverse events must be recorded annually.

Examination by HRCT may cause discomfort in the form of claustrophobia in some patients. In addition, a CT scan involves exposure to rays equal to approximately 5 years of background radiation in Denmark. As the study participants only will be examined once, and as the median life expectancy for this group of patients with severe COPD is approximately 5 years, the diagnostic benefit it is estimated to be greater than the risk of exposure.

## 8. Removal from and interruption of the trial

Investigators may interrupt the intervention at any time if there is a medical justification, safety risk or a requirement from the authorities. If the investigator deems it necessary, he or she may exclude the participant from the trial. However, in general, no subject should be removed from the study for a protocol violation prior to confirmation by the coordinating investigator. In addition, a study participant is only to be withdrawn from the study if the participant explicitly asks for withdrawal.

## 9. Funding

The research project is financed by grants from the Independent Research Fund Denmark (8020-00425B) and the Research committee at Herlev and Gentofte University Hospital and by participating study centres.

## 10. Access to data

It is the Steering Committee's belief that sharing of knowledge creates more and better scientific results. Requests for sharing data with other groups will be submitted to the Steering Committee. If the hypothesis to be examined has a relevant scientific content, and is not planned to be examined by our group, we will allow the use of our data

## 11. Publication of study results

The trial registered at clinicaltrials.gov (NCT03262142, August 25, 2017). The data from the TARGET ABC trial will be available once the study is completed. All results will be published in scientific contexts, including international journals, regardless of whether they are positive, negative or in-conclusive, and with authorship according to the Vancouver recommendations.

## 12. Scientific ethical statement

*P. aeruginosa* represents a potentially significant cause of exacerbation and mortality in patients with COPD, non-CF bronchiectasis, and asthma. However, the role of *P. aeruginosa* in this setting is poorly characterized, and to date, evidence-based guidelines for management and treatment of *P. aeruginosa* infection are lacking. With this trial, we aim to increase the knowledge of the clinical consequences of antibiotic treatment against *P. aeruginosa* in patients with COPD, non-CF bronchiectasis and asthma.

Using a multicenter, randomized, controlled design, we will allocate 150 patient with COPD, non-CF bronchiectasis or asthma and current *P. aeruginosa*-positive respiratory sample (1:1) to either no antibiotic treatment or 14 days of dual anti-pseudomonal antibiotic therapy. Thus, we seek to create evidence at level 1B to determine whether targeted antibiotic treatment against *P. aeruginosa* can reduce exacerbations in COPD, non-CF bronchiectasis and asthma and thereby improve the prognostic outcome in a group of severe and vulnerable patients with chronic lung disease.

The trial is carried out in accordance to the Declaration of Helsinki and follows Good Clinical Practice. Study methods and statistical analyses have been carefully considered and it is our strongest belief, that the trial will contribute with essential knowledge that will help clinicians to guide future patients towards evidence-based and improved therapeutically strategies. In addition, the likelihood of serious adverse reactions to the antibiotics is expected to be low and the investigator can always interrupt treatment if it is considered contraindicated. Patients who do not wish to participate in the trial and study participants who withdraw their informed consent will be offered treatment according to the standard guidelines at the specific study department. Based on the above considerations, we believe that the trial is ethically sound and can be conducted without exposing the study participants to unjustifiable risks.

## 13. Protocol references

1. Soriano JB, Abajobir AA, Abate KH, Abera SF, Agrawal A, Ahmed MB, et al. Global, regional, and national deaths, prevalence, disability-adjusted life years, and years lived with disability for chronic obstructive pulmonary disease and asthma, 1990–2015: a systematic analysis for the Global Burden of Disease Study 2015. Lancet Respir Med. 2017;5(9):691–706.

2. Henkle E, Chan B, Curtis JR, Aksamit TR, Daley CL, Winthrop KL. Characteristics and Health-care Utilization History of Patients With Bronchiectasis in US Medicare Enrollees With Prescription Drug Plans, 2006 to 2014. Chest. 2018;154(6):1311–20.

3. Jacobs DM, Ochs-Balcom HM, Noyes K, Zhao J, Leung WY, Pu CY, et al. Impact of Pseudomonas aeruginosa isolation on mortality and outcomes in an outpatient chronic obstructive pulmonary disease cohort. Open Forum Infect Dis. 2020;

4. GOLD. Global intiative for Chronic Obstructive Lung Disease 2020 Report: pocket guide to COPD daignosis, management, and prevention, a guide for health care professionals. GOLD 2020. 2020. https://goldcopd.org.

5. Global Initiative for Asthma: 2019 GINA Report. Global strategy for asthma management and prevention. [https://ginasthma.org/wpcontent/uploa ds/2019/06/GINA-2019-main-report-June-2019-wms.pdf](https://ginasthma.org/wpcontent/uploa%20ds/2019/06/GINA-2019-main-report-June-2019-wms.pdf).

6. Polverino E, Goeminne PC, McDonnell MJ, Aliberti S, Marshall SE, Loebinger MR, et al. European Respiratory Society guidelines for the management of adult bronchiectasis. European Respiratory Journal. 2017.

7. Quint JK, Millett ERC, Joshi M, Navaratnam V, Thomas SL, Hurst JR, et al. Changes in the incidence, prevalence and mortality of bronchiectasis in the UK from 2004 to 2013: A population-based cohort study. Eur Respir J. 2016;47(1):186–93.

8. Tuon FF, Dantas LR, Suss PH, Tasca Ribeiro VS. Pathogenesis of the Pseudomonas aeruginosa Biofilm: A Review. Pathogens. 2022.

9. Garcia-Vidal C, Almagro P, Romaní V, Rodríguez-Carballeira M, Cuchi E, Canales L, et al. Pseudomonas aeruginosa in patients hospitalised for COPD exacerbation: A prospective study. Eur Respir J. 2009;

10. Groenewegen KH, Wouters EFM. Bacterial infections in patients requiring admission for an acute exacerbation of COPD; a 1-year prospective study. Respir Med. 2003;

11. Patel IS, Seemungal TAR, Wilks M, Lloyd-Owen SJ, Donaldson GC, Wedzicha JA. Relationship between bacterial colonisation and the frequency, character, and severity of COPD exacerbations. Thorax. 2002;

12. Borekci S, Halis AN, Aygun G MB. Bacterial colonization and associated factors in patients with bronchiectasis. Ann Thorac Med. 2016;11(1):55–9.

13. Holm JPY, Hilberg O, Noerskov-Lauritsen N, Bendstrup E. Pseudomonas aeruginosa in patients without cystic fibrosis is strongly associated with chronic obstructive lung disease. Dan Med J. 2013;

14. Miravitlles M. Relationship Between Bacterial Flora in Sputum and Functional Impairment in Patients With Acute Exacerbations of COPD. CHEST J. 1999;116(1):40–6.

15. Sangtam N, Haorongbam S, Silpa K, Singh YP. Bronchiectasis in patients with chronic obstructive pulmonary disease in a tertiary care center in North-East India. Int J Adv Med. 2020;

16. Folkesson A, Jelsbak L, Yang L, Johansen HK, Ciofu O, Hoiby N, et al. Adaptation of Pseudomonas aeruginosa to the cystic fibrosis airway: An evolutionary perspective. Nat Rev Microbiol. 2012;10(12):841–51.

17. Guidelines for antibiotic treatement of acute pulmonary infection with Pseudomonas aeruginosa in COPD. Capital Region, Denmark.

18. Chalmers JD, Goeminne P, Aliberti S, McDonnell MJ, Lonni S, Davidson J, et al. The bronchiectasis severity index an international derivation and validation study. Am J Respir Crit Care Med. 2014;189(5):576–85.

# STATISTICAL ANALYSIS PLAN

**Targeted AntiBiotics for Chronic pulmonary diseases (TARGET-ABC)**

**A multicenter, randomized, controlled, open-label trial**

**ClinicalTrials.org Identifier:** NCT03262142

**Author:** Josefin Eklöf, Jens-Ulrik Stæhr Jensen

**Introduction:**

This is a multicenter, randomized, controlled, open-label trial evaluating the effect of antibiotic treatment for *P. aeruginosa* in patients with chronic pulmonary disease.

The aim of the study is to investigate whether targeted antibiotics against *P. aeruginosa* can reduce exacerbations and mortality in patients with chronic obstructive pulmonary disease (COPD), non-CF bronchiectasis (non-CF BE) and asthma.

The patients are enrolled in the trial only after obtaining informed consent. The trial is conducted at seven centers in Denmark:

**1.** Department of Internal Medicine, Section of Respiratory Medicine, Herlev-Gentofte Hospital, University of Copenhagen.

Primary investigator: Josefin Eklöf, MD, PhD.

**2**. Department of Pulmonary and Infectious Diseases, University Hospital North Zealand Hospital. Primary investigator: Andrea Browatzki, MD.

**3.** Department of Respiratory Medicine, Amager-Hvidovre Hospital, University of Copenhagen.

Primary investigator: Julie Janner, MD, PhD.

**4.** Department of Respiratory Medicine, Bispebjerg-Frederiksberg Hospital, University of Copenhagen.

Primary investigator: Therese Lapperre, MD, PhD, Research Associate Professor.

**5.** Department of Respiratory Medicine, Aalborg Hospital, University of Aalborg.

Primary investigator: Ulla Weinreich, MD, PhD, Research Associate Professor.

**6.** Department of Respiratory Medicine, Odense University Hospital.

Primary investigator: Sofie Johansson, MD, PhD

**7.** Department of Internal Medicine, Section of Respiratory Medicine, Hospital of Southwest Jutland, Esbjerg.

Primary investigator: Torben Tranborg Jensen, MD.

Patients will be randomized 1:1 to one of the two treatment arms:

1. **Intervention group**: intravenous beta-lactam in combination with oral ciprofloxacin for 14 days
2. **Control group**: no antibiotic treatment

The analyses described in this document will be performed by the coordinating investigator, Josefin Eklöf, in cooperation with the scientific sponsor, Jens Ulrik Stæhr Jensen, once the data have been entered, cleaned, and released for use.

This statistical analysis plan provides a detailed description of the statistical analyses that will be performed for the evaluation of the primary and secondary endpoints of the TARGET-ABC study.

The analyses described in this document are compatible with the recommendations of the CONSORT 2010 statement.

**Analyses:**

Data will be analysed using intention-to-treat (ITT) principles, including all the data available, regardless of whether the participant completed the intervention or not. The aim of the ITT analysis is also to provide unbiased comparisons among the two study groups and to avoid the effects of potential study dropouts and protocol deviations. The primary outcome will also be subject to a modified ITT analysis (in study participants who started but did not complete the intervention) and per protocol analysis (in study participants who

completed intervention).

A Consort diagram of participants will be presented in the study.

Patients who withdraw their consent for the use of their data will not be included in any analysis. Patients who merely withdraw consent to the intervention, will be included in the ITT and modified ITT analysis. We will report cases of withdrawal and the study group to which the participant was originally allocated.

All analyses will be performed using SAS software.

**Sample size:**

The power to avoid type II error is 80% (1-β) at a two-sided 5% significance level. We used a group-sequential design, allowing for one interim analysis at half target recruitment. This provides a sample size of 150 subjects. All confidence intervals reported will be 95% confidence intervals.

**Descriptive analyses:**

The following baseline characteristics will be presented within each randomized study group:

- - - - Age, years, median (IQR)
      - Male sex, n (%)
      - Ethnicity (Caucasian, African (incl. Afro-American), Asian, Inuit, Unknown/other), n (%)
      - Body mass index (kg/m2), median (IQR)
      - Medical Research Council dyspnea scale, n (%)
      - Current smoking, n (%)
      - Former smoking, n (%)
      - Non-smoking, n (%)
      - Pack-years tobacco history, median (IQR)
      - COPD assessment test score (CAT), median (IQR)
      - Support with activities of daily living at home, n (%)
      - Increased dyspnea, n (%)
      - Increased sputum volume, n (%)
      - Increased sputum purulence, n (%)
      - Increased cough, n (%)
      - Systolic blood pressure (mm Hg), median (IQR)
      - Diastolic blood pressure (mm Hg), median (IQR)
      - Heart rate, beats/min, median (IQR)
      - Oxygen saturation with nasal oxygen, median (IQR)
      - Respiratory rate, breaths/min, median (OQR)
      - Temperature (°C), median (IQR)
      - Exacerbation frequency in previous year, median (IQR)
      - Current or former use of respiratory medication, including antibiotics, n (%)
      - Use of long-term oxygen therapy, n (%)
      - Use of noninvasive mechanical ventilation, n (%)
      - Co-morbidities, n (%)
      - FEV1, L, median (IQR)
      - FEV1 % predicted, median (IQR)
      - FVC, L, median (IQR)
      - FVC % predicted, median (IQR)
      - FEV1/FVC ratio, %, median (IQR)

**Follow-up data /missing data**

We expect the extent of missing data to be small in the current trial, and we do not expect that any patients will be lost to follow-up for the primary endpoint.

Following measures will be used in case of missing data:

1. For each baseline variable, the percent of any missing values will be reported.
2. The proportion of patients followed for each outcome data parameter will be reported for the predefined primary and secondary outcomes – as well as in any potential exploratory outcome analyses suggested by external reviewers or editors.
3. Characterization participants for whom no outcomes were observed.
4. Report possible reasons for missing outcome data.
5. We will perform sensitivity analysis to quantify the effect of missing outcome data using multiple imputation on study results

**Primary objective and outcomes**

The primary outcome is *

1. Time to prednisolone and/or antibiotic requiring exacerbation, in primary or secondary health care sector, or death from day 20 to day 365 from randomisation

The primary outcome will also be analysed as an adjusted analysis using a multivariable Cox proportional hazards model, adjusting for the following variables: sex (male vs. female), CAT-score at recruitment (< 21 vs. ≥ 21) and FEV1 % predicted at recruitment (<50% vs. >= 50%).

*The co-primary outcome of "days alive and without hospitalisation from day 20 to day 365 from randomisation" was degraded to the first secondary outcome by the trial statistician in agreement with the coordination investigator and study director since this outcome would be severely underpowered because of the premature closure of the trial. This decision was taken before the database was unblinded to the analysis (see Supplementary page x).

**Secondary objective and outcomes**

1. Days alive and without hospitalisation from day 20 to day 365 from randomisation

Analysis: t-test or Mann-Whitney U test

1. Death within 365 days from randomisation

Analysis: Fisher's exact test or Chi squared test

1. Number of re-admissions with pulmonary exacerbation within 365 days from randomization

Analysis: t-test or Mann-Whitney U test

1. Number of days with non-invasive-ventilation or invasive ventilation within 90 days from randomization

Analysis: t-test or Mann-Whitney U test

1. Microbiological cure (defined as *P. aeruginosa*-negative sputum culture until day 90)

Analysis: Fisher's exact test or Chi squared test

1. Clinical cure day 14 (defined as cessation or improvement of clinical signs and symptoms related to *P. aeruginosa* before or on day 14)

Analysis: Fisher's exact test or Chi squared test

1. Change in COPD Assessment Test (CAT) from randomization to day 90

Analysis: ANOVA will be used to analyse the difference between the two means

1. Change in body mass index (BMI) from randomization to day 90

Analysis: ANOVA will be used to analyse the difference between the two means

1. Change in FEV_1_ from randomization to day 90

Analysis: ANOVA will be used to analyse the difference between the two means

1. Decrease of ≥ 200 ml in FEV_1_ from randomization to day 365

Analysis: Fisher's exact test or Chi squared test

**Interim Analysis:**

The interim analysis will focus on reporting:

1. Baseline characteristics
2. Primary outcome: Time to prednisolone and/or antibiotic requiring exacerbation or death, in primary or secondary health care sector, from day 20 to day 365 from randomization (using O- Brien-Fleming Plot)
3. All-cause mortality at 365 days
4. Microbiological cure
5. Futility assessment. Recruitment rate compared to planned recruitment.

**Blinding of the statistician**

The detailed analysis plan was written in strict concordance with the trial protocol approved by the regulatory authorities prior to recruitment initiation. The entire statistical analysis plan is published at www.coptrin.dk (before the trial was finalized and while the database was closed). All analyses will be done prior to breaking of the randomization code (analysis comparisons between “arm A” and “arm B”). The coordinating investigator and the study sponsor and principal investigator will conjointly perform all the data analyses according to this plan, except the interim analysis, which will be performed by a statistician who is not an investigator in the trial. An unblinding date will be chosen and published online at www.coptrin.dk and on this date, the allocation will be unblinded. After the unblinding, no further analysis will not be done, except on demand of reviewer or editor during the publication process.

**Figures and tables**

The first figure will be a Consolidated Standards of Reporting of Randomized Trials (CONSORT) flow chart. The second figure will be a Kaplan-Meier plot to describe the process of death by treatment arms. The first table will be the baseline characteristics of the ITT population. The second table will be of the primary and secondary outcomes according to the two groups and pair-wise comparisons.

**Interim and final analysis**

The interim analysis was planned at half target recruitment (75 patients), with a focus on reporting data on the primary outcome (time to prednisolone and/or antibiotic requiring exacerbation or death at day 20 to day 365 from randomisation), all-cause mortality at day 365, microbiological cure at day 14 and assessment of the study’s futility. An independent data and safety monitoring board (DSMB) was appointed to review the trial’s safety, efficacy, and progression (Appendix page 18: DSMB members and charter). Due to slow recruitment rate, the steering committee of the trial decided to stop further recruitment in February 2022, where approximately 1/3 of the planned patients had been enrolled in the study (Appendix page 3: Study timeline). This decision was tested with the DSMB, who endorsed it (Appendix page 23). Due to the considerable reduction of the study size, the primary outcome was conducted solely as a “time to event” analysis, and the “days alive and out of hospital” analysis was degraded from a co-primary outcome to the first secondary outcome (please see under “Outcomes”). Data analyses were performed by an analysis team (TWK and AJ), including a trial statistician (TWK), after the final data from the last patients last follow-up visit was entered and the database was locked. All analyses were done prior to breaking of the randomisation code. The study group was presented to the results and unblinded at a scheduled unblinding-meeting after the analyses were performed.

# RANDOMISATION AND MASKING

The randomisation sequence was generated using a computed generator, stratified according to study site and age (≤70 years vs. >70 years). Online inclusion of patients according to the concealed sequence was done with an independent, centralised, 24 hour-available, web-based system (Redcap). The randomisation sequence was prepared by PS, with instructions from the study director (JUSJ), who did not participate in the enrolment of patients in the trial, nor outcome assessment. Since the study was open-label and without a placebo, both investigator, staff and patients were aware of the allocation to either the control arm or antibiotic intervention from the time of randomisation.

# ADHERENCE TO TREATMENT

|  | Antibiotic intervention group, n (percent) |
| --- | --- |
| Patients who did not adhere to the study protocol and received no dual systemic antibiotics | 1 (3·8) |
| Patients who were partially adherent and received 1 to 13 days of dual systemic antibiotics | 1 (3·8) |
| Total | 2 (7·7) |

|  | Control group, n (percent) |
| --- | --- |
| Patients who were partially adherent and received 1 to 13 days of pseudomonas active antibiotics | 1 (4·3) |
| Patients who were fully non-adherent and received 14 days of pseudomonas active antibiotics | 0 (0·0) |
| Total | 1 (4·3) |

# PROTOCOL AMENDMENT LOG

| **Date** | **Description of changes** |
| --- | --- |
|  |  |
| 2 March 2018 | Specification of exacerbation-inclusion criteria: exacerbation of COPD treated with prednisolone or/and antibiotics |
| 5 January 2019 | Change of antibiotic intervention. Standard dose regiment of piperacillin/tazobactam changed from three to four times daily according to regional guidelines (9 November 2018 and EUCAST) |
| 21 October 2019 | Added Esbjerg Hospital as trial site |
| 24 January 2020 | Change of inclusion criteria: Changed from requiring diagnosis with COPD to requiring diagnosis with COPD or/and asthma or/and non-cystic fibrosis bronchiectasis.  Change of intervention. Added possibility of home treatment. |

# DATA AND SAFETY MONITORING BOARD CHARTER

**TARGET ABC trial:**

**Targeted AntiBiotics for Chronic pulmonary diseases**

**-**

**A multicenter, randomized, controlled, open-label trial**

**Study identification information**

- 1. **Sponsors protocol code:** TARGET ABC (ClinicalTrials.org Identifier: NCT03262142)
  2. **Study title:** Targeted antibiotics for chronic pulmonary diseases
  3. **Sponsor:** Jens-Ulrik Stæhr Jensen, MD, PhD, professor, Department of Internal Medicine, Section of Respiratory Medicine, Copenhagen University Hospital - Gentofte, Hellerup, Denmark.
  4. **Coordinating investigator:** Josefin Eklöf, MD, PhD, Department of Internal Medicine, Section of Respiratory Medicine, Copenhagen University Hospital - Gentofte, Hellerup, Denmark.
  5. **Study centers:**

1. Department of Internal Medicine, Respiratory Medicine Section, Herlev and Gentofte Hospital, Denmark

2. Department of Respiratory Medicine and Infectious Medicine, North Zealand Hospital, Denmark

3. Department of Respiratory Medicine and Infectious Diseases, Bispebjerg Hospital, Denmark

4. Department of Respiratory Medicine, Amager and Hvidovre Hospital, Denmark

**5.** Department of Respiratory Medicine, Aalborg Hospital, University of Aalborg

6. Department of Respiratory Medicine, Odense University Hospital.

7. Department of Respiratory Medicine, Southwest Jutland Hospital, Esbjerg, Denmark

- 1. **Members of the DSMB:**

The following members have been requested to be part of DSMB:

- Tor Biering Sørensen, MD, Ph.d., MPH
  - - - Dept of Cardiology, Gentofte University Hospital, Hellerup, Denmark
- Philipp Schuetz, Professor, Dr.Med. MPH
  - - - Kantonspital Aarau AG |
      - KSA · Internal Medicine & Emergency Medicine
- Tobias Wirenfeldt Klausen, MSc, trial statisticians

The independent Data and Safety Monitoring Board (DSMB) is established to ensure the safety of research participants and the integrity of the study data. It will periodically monitor progress, efficacy, safety and other confidential data from this trial. It is composed of experts in relevant medical fields who have no direct relationship with the study. Outcome data will be privileged and shared only with members of the DSMB during the conduct of the trial.

**Study Overview**

This study is a multicenter, randomized, controlled, open-label trial. A total of 150 patients with COPD, non-CF bronchiectasis or asthma, and *P. aeruginosa*-positive lower respiratory tract samples, will be randomly assigned with a 1:1 ratio to either no antibiotic treatment (control group) or anti-pseudomonal antibiotic treatment with intravenous beta-lactam and oral ciprofloxacin (intervention group) for 14 days.

The monitoring guideline outlined below will adhere to the protocol approved by the Ethics Committees of all participating sides (H-15010949), Danish Medicines Agency (EudraCT:2015-003399-58) and the Danish Data Protection Agency.

**Data Quality and Safety Review Plan and Monitoring**

- 1. **Subject Recruitment**

Review of the rate of subject accrual and compliance with inclusion/exclusion criteria will occur regularly to ensure that a sufficient number of participants are being enrolled and meet eligibility criteria. Recruitment rate will be reported by a recruitment graph.

- 1. **Justification of Sample Size**

We expect an effect size of 20% absolute reduction (30% relative reduction) of exacerbation or mortality in the antibiotic group:

- Sixty-seven percent of study participants in the antibiotic-free group have exacerbated or died within 12 months
- Forty-seven percent of study participants in the antibiotic group have exacerbated or died within 12 months

The power to avoid type II error is 80% (1-β) at a two-sided 5% significance level. We used a group-sequential design, allowing for one interim analysis at half target recruitment. Based on these estimates and indicative figures, a total of 150 patients should be included (i.e., 75 patients in each group).

- 1. **Stopping Rules**

This study will be stopped prior to its completion if the intervention is associated with adverse effects that call into question the safety of the intervention or failure of feasibility.

- 1. **Designation of an Independent Monitor**

The Independent Monitor for this study is the GCP (Good Clinical Practice)-unit at Copenhagen University Hospital.

- 1. **Safety Review**

The DSMB review will focus:

i) Baseline characteristics

ii) Adherence data

iii) Follow-up (missing data)

iv) Outcome data

v) Accrual data (data for feasibility for completion)

We have planned the interim analysis when all the data from the first 75 patients have been entered into the database (half of the patients recruited). The DSMB may, apart from this planned interim analysis, decide to request an extra-ordinary interim analysis at any time point. This will be blinded to the investigators.

**Analyses**

The analyses described in this document will be performed in a blinded manner (“group A” and “group B”) by a trial statistician with guidance from the sponsor, once the data have been entered, cleaned and released for use.

This document provides a description of the statistical analyses that will be performed for the evaluation of the primary and secondary endpoints of the TARGET ABC trial. The analyses described in this document are compatible with the recommendations of the CONSORT 2010 statement.

1. **Analysis population**

Data will be analyzed using intention-to-treat (ITT) principles. All randomized patients will be analyzed in the groups to which they were originally allocated to, regardless of whether they actually received the intended treatment or whether a protocol violation or deviation occurred.

Patients who withdrew consent for use of their data will not be included in any analysis. Only the facts that they were enrolled into the trial, including the study group to which they were allocated, and that they withdrew consent, will be reported. Patients who withdraw consent to the intervention, but allowed for data analysis, will be included in the ITT as well as a modified ITT analysis.

Two-sided 5% significance levels will be used to identify statistically significant results. All confidence intervals reported will be 95% confidence intervals. All analyses will be performed using SAS software version 9.4.

1. **Definitions**

Baseline: day 1 (randomisation)

Follow-up visits: 14, 30, 60, 90 and 365 days.

1. **Baseline characteristics**

The following baseline characteristics of the study population will be summarized separately within each randomized group:

- Age, median (interquartile range)
- Male, n (%)
- Body mass index, median (interquartile range)
- Smoking status: current smoker, ex-smoker, never-smoker, n (%)
- Pack-years history, median (interquartile range)
- FEV1 % predicted, median (interquartile range)
- Use of long-term oxygen treatment (LTOT), n (%)
- Home-NIV, n (%)

For each variable, the percent of missing values will be reported.

##

1. **Adherence data**

Adherence to the antibiotic intervention will be evaluated by computer registered information on administered antibiotics and medicine log filled by the participants. Adherence will be reported as good (> 60%), partial (40-60%), and non-adherent (< 40%).

1. **Follow-up (missing data)**

The proportion of patients followed for each outcome data parameter will be reported for the predefined primary and secondary outcomes. Possible reasons for missing outcome data will be reported.

1. **Outcome data (for the pre-planned interim analysis)**

The interim analysis will be performed, when we have included 50% of the sample size (75 patients)

- Primary outcome:

Co-primary outcome: i) Time to prednisolone and/or antibiotic requiring exacerbation or death, in primary or secondary health care sector, from day 20 to day 365 from randomization ii) Days alive and without hospitalisation from day 20 to day 365 from randomisation ("Days alive and without hospitalisation from day 20 to day 365 from randomisation" was degraded to the first secondary outcome by the trial statistician in agreement with the coordination investigator and study director since this outcome would be severely underpowered because of the premature closure of the trial. This decision was taken before the database was unblinded to the analysis (see Supplementary page 24).

- Secondary outcome: All-cause mortality at 365 days
- Secondary outcome: Microbiological cure at 90 days
- Explaining data

1. **Accrual data (data for feasibility for completion)**

Recruitment rate will be compared to the planned recruitment in order to assess the futility of the trial.

Data will be presented as the total number of recruited patients per site.

#

# PATIENT RECRUITMENT PER TRIAL SITE


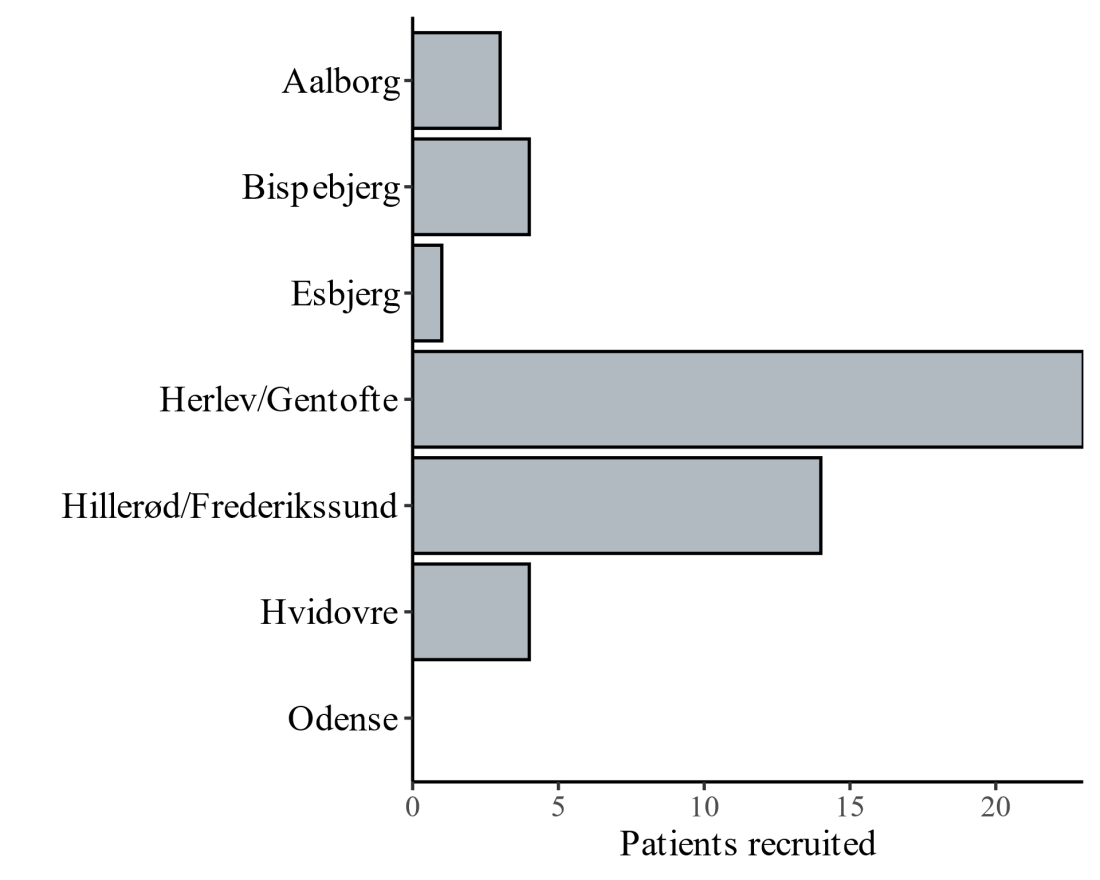


# DSMB ENDORSMENT LETTER FOR EARLY TERMINATION


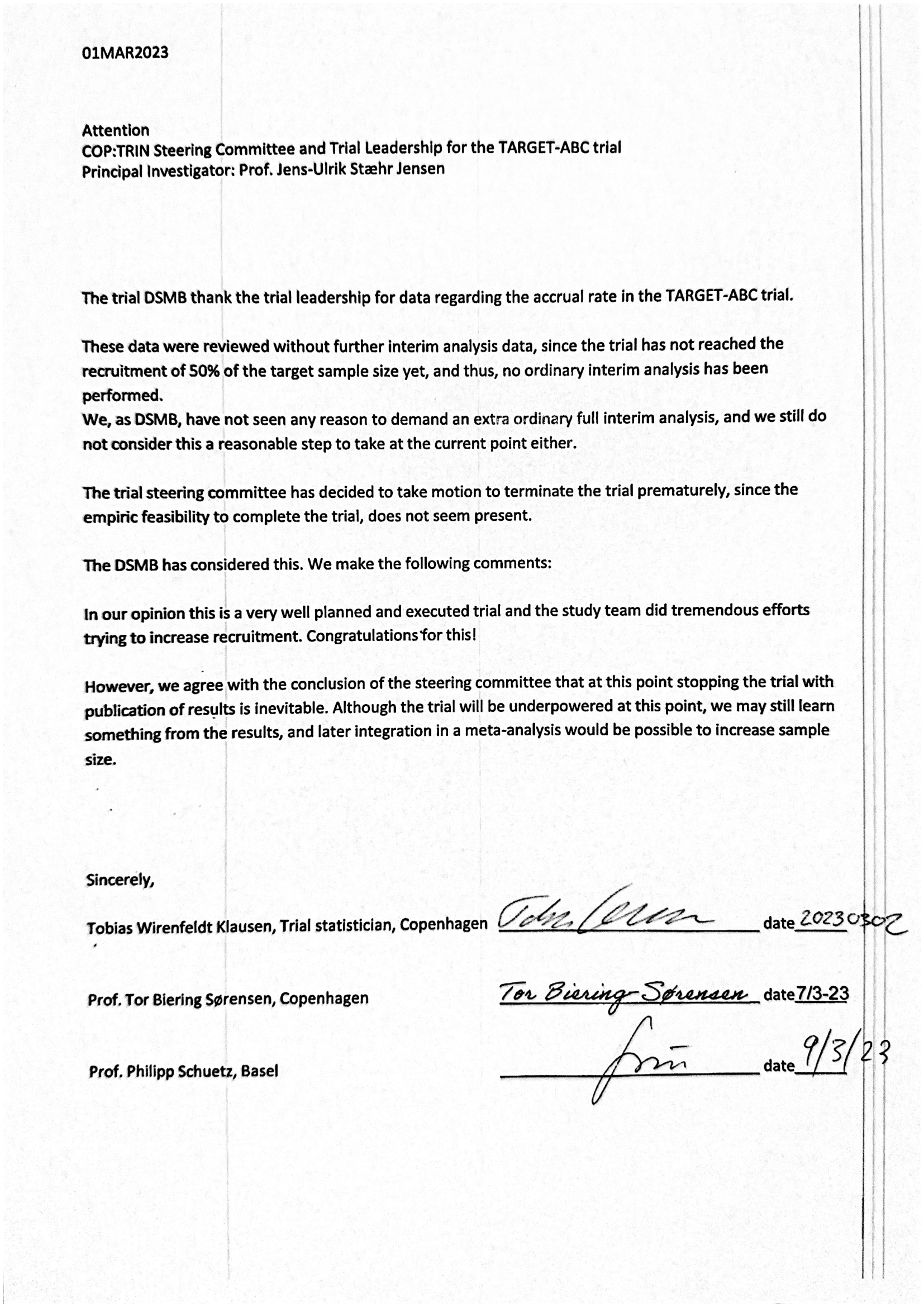


# NOTE TO STATISTICAL ANALYSIS PLAN PRIOR TO DATABASE UNLOCKING


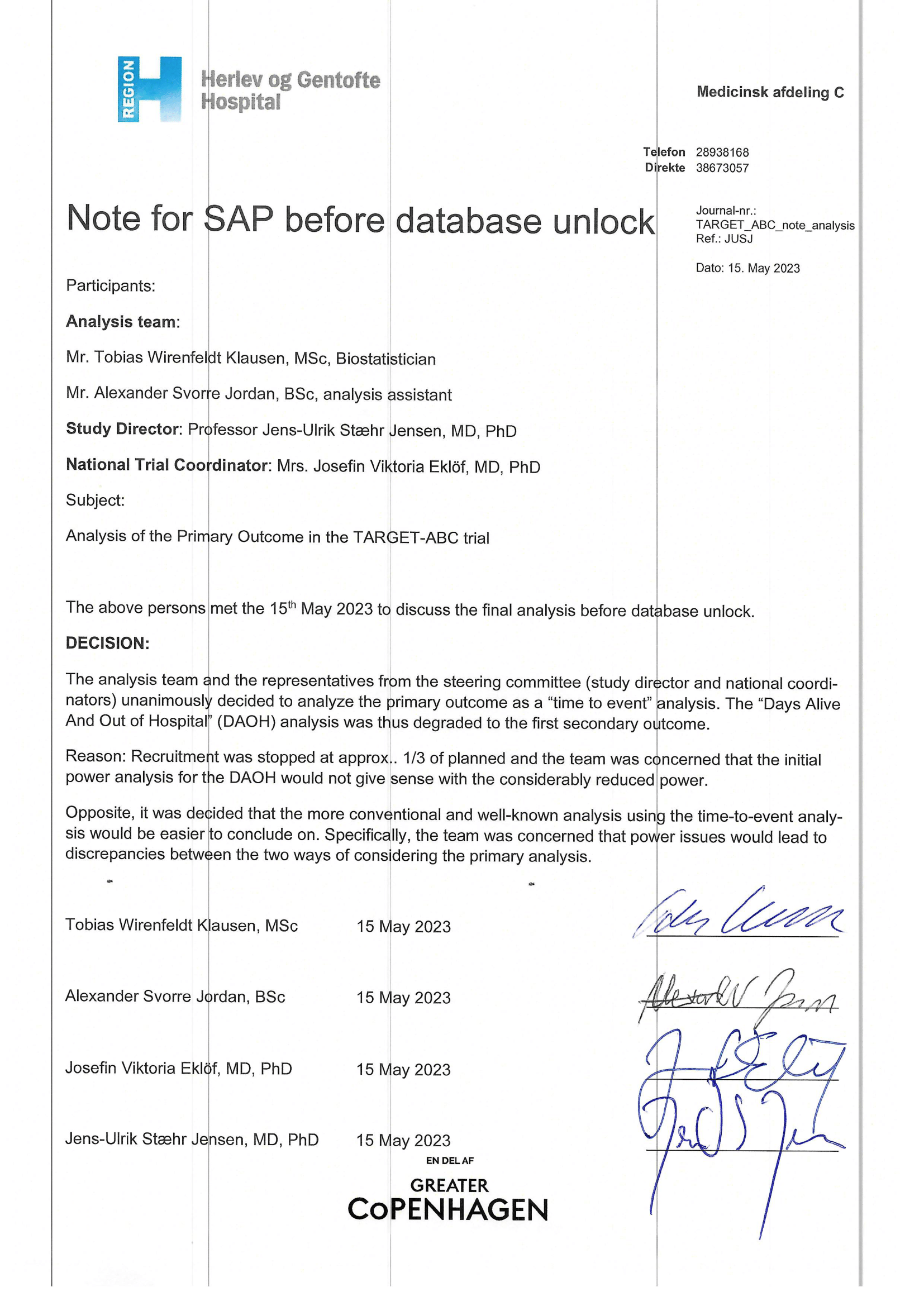


# SUPPLEMENTAL TABLES AND FIGURES

**Table 1: Cox regression for primary endpoint, exacerbation or death within days 20 to 365 in the ITT population for selected patient subgroups.**

|  | | **Antibiotic group** | **Control group** |  | |
| --- | --- | --- | --- | --- | --- |
|  | | **N events (%)** | **N events (%)** | **Crude HR (95% CI)** | **P value** |
|  | Patients with COPD (n=40) | 13 (68) | 20 (95) | 0·46 (0·23—0.93) | 0·030 |
|  | Patients with asthma (n=11) | 5 (63) | 3 (100) | 0·75 (0.18—3·19) | 0·70 |
|  | Patients with bronchiectasis (n=20) | 7 (58) | 8 (100) | 0·37 (0·13—1·03) | 0·056 |

**Table 2. Number of patients with *Pseudomonas aeruginosa*-positive sputum samples during the planned study visits.**

|  | **Day 14** | **Day 30** | **Day 60** | **Day 90** | **Day 360** |
| --- | --- | --- | --- | --- | --- |
| **Study group** |  |  |  |  |  |
| Antibiotic group | 1 | 8 | 9 | 12 | 5 |
| Control group | 15 | 11 | 8 | 12 | 6 |
| Total in both study groups | 16 | 19 | 17 | 24 | 11 |

**Table 3: Post-hoc analyses for comparing changes in selected secondary outcome measures at other time points.**

|  | **Antibiotic group (n = 26)** | **Control group (n = 23)** | **Difference (95% CI)** | **P value** | **Number missing** |
| --- | --- | --- | --- | --- | --- |
| Change in CAT from baseline to day 30 day, mean (95% CI) | -4·0 (-6·8—-1·1) | -2·5 (-4·8—-0·1) | -2·2 (-5·7—1·3) | 0·21 | 7 |
| Change in CAT from baseline to day 365, mean (95% CI) | -1·8 (-5·0—1·4) | -1·9 (-5·7—1·8) | -0·9 (-5·4—3·6) | 0·69 | 12 |
| Change in FEV1 (L) from baseline to day 30 day, mean (95% CI) | 0·08 (0·01—0·15) | -0·03 (-0·08—0·02) | 0·11 (0·03—0·19) | 0·011 | 7 |
| Change in FEV1 (L) from baseline to day 365, mean (95% CI) | 0·06 (-0·04—0·16) | -0·03 (-0·10—0·04) | 0·09 (-0·03—0·21) | 0·14 | 18 |

CAT=COPD assessment test. FEV_1_=forced expiratory volume the first second.

**Table 4: Adverse events in the study population**

|  | | **Antibiotic group (n = 26)** | **Control group (n = 23)** | **P value** |
| --- | --- | --- | --- | --- |
| **Severe adverse events** | |  |  |  |
|  | Infections and parasitic diseases, n (%) | 1 (4) | 0 (0) |  |
|  | Metabolism and nutrition, n (%) | 0 (0) | 1 (4) |  |
|  | Airways, thorax and mediastinum, n (%) | 1 (4) | 1 (4) |  |
|  | Kidneys and urinary tract, n (%) | 1 (4) | 0 (0) |  |
|  | Any severe adverse event, n (%) | 3 (12) | 1 (4) | 0·61 |
| **Non-severe adverse events** | |  |  |  |
|  | Blood and lymphatic system, n (%) | 0 (0) | 4 (17) |  |
|  | Immune system, n (%) | 1 (4) | 0 (0) |  |
|  | Heart, n (%) | 2 (8) | 0 (0) |  |
|  | Airways, thorax and mediastinum, n (%) | 0 (0) | 1 (4) |  |
|  | Gastrointestinal tract, n (%) | 1 (4) | 0 (0) |  |
|  | Skin and subcutaneous tissues, n (%) | 1 (4) | 0 (0) |  |
|  | Bones, joints, muscles, and connective tissues, n (%) | 1 (4) | 1 (4) |  |
|  | Kidneys and urinary tract, n (%) | 1 (4) | 1 (4) |  |
|  | Any non-severe adverse event, n (%) | 6 (23) | 5 (22) | 1·00 |
| **Any adverse event, n (%)** | | 9 (34) | 6 (26) | 0·55 |

**Figure 3: Days alive and out of hospital from day 20 to 365**


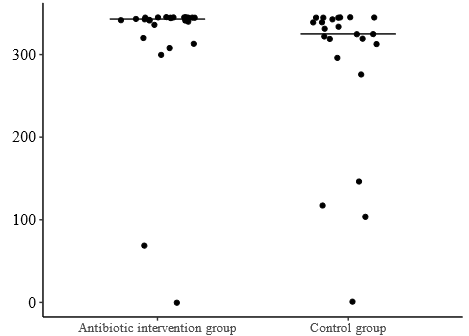


**Figure 4. Mortality within 365 days**


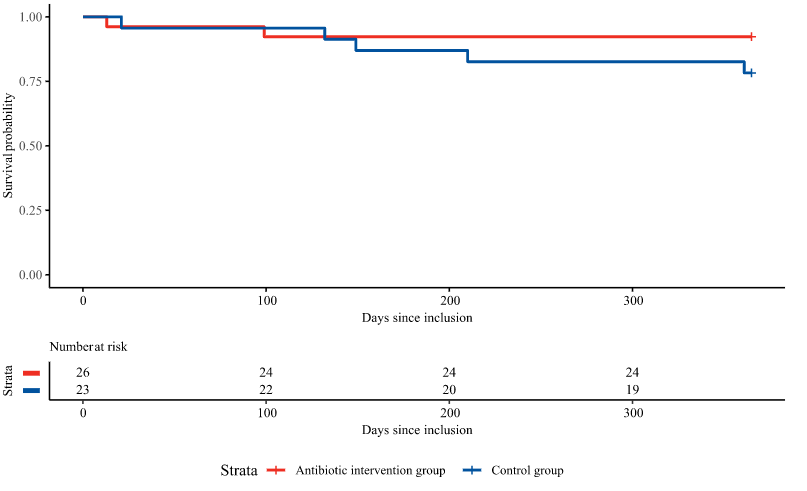


**Figure 5. Number of hospital admissions with exacerbation within 365 days from randomization**


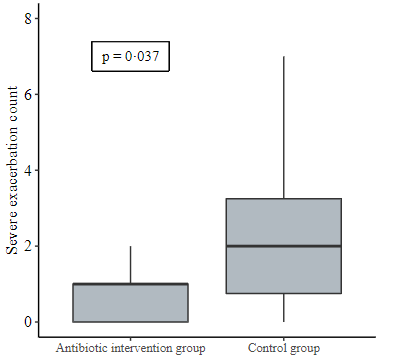


**Figure 6. Changes in CAT scores (A), body mass index (B), and FEV_1_ (C) from randomization to day 365**


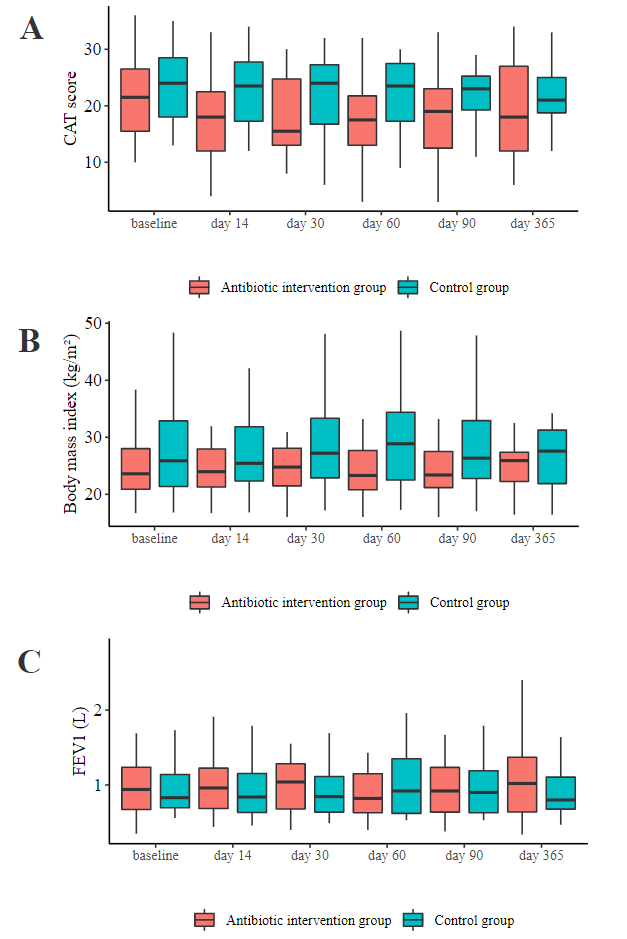

Supplement: Supplementary file 1 — Additional file 1: 1. Trial group. 2. Study timeline. 3. Study protocol. 4. Statistical analysis plan. 5. Randomisation and masking. 6. Adherence to treatment. 7. Protocol amendment log. 8. Data and Safety Monitoring Board (DSMB) charter. 9. Patient recruitment by trials site. 10. DSMB endorsement letter for early trial termination. 11. Note to statistical analysis plan. 12. Supplemental tables and figures. [file 12931_2024_2860_MOESM1_ESM.docx]
